# SePIA: RNA and small RNA sequence processing, integration, and analysis

**DOI:** 10.1186/s13040-016-0099-z

**Published:** 2016-05-20

**Authors:** Katherine Icay, Ping Chen, Alejandra Cervera, Ville Rantanen, Rainer Lehtonen, Sampsa Hautaniemi

**Affiliations:** Research Programs Unit, Genome-Scale Biology, Medicum and Department of Biochemistry and Developmental Biology, Faculty of Medicine, University of Helsinki, POB 63, Helsinki, 00014 Finland

**Keywords:** Sequencing, Integration, miRNA, totalRNA, RNA, Breast cancer

## Abstract

**Background:**

Large-scale sequencing experiments are complex and require a wide spectrum of computational tools to extract and interpret relevant biological information. This is especially true in projects where individual processing and integrated analysis of both small RNA and complementary RNA data is needed. Such studies would benefit from a computational workflow that is easy to implement and standardizes the processing and analysis of both sequenced data types.

**Results:**

We developed SePIA (Sequence Processing, Integration, and Analysis), a comprehensive small RNA and RNA workflow. It provides ready execution for over 20 commonly known RNA-seq tools on top of an established workflow engine and provides dynamic pipeline architecture to manage, individually analyze, and integrate both small RNA and RNA data. Implementation with Docker makes SePIA portable and easy to run. We demonstrate the workflow’s extensive utility with two case studies involving three breast cancer datasets. SePIA is straightforward to configure and organizes results into a perusable HTML report. Furthermore, the underlying pipeline engine supports computational resource management for optimal performance.

**Conclusion:**

SePIA is an open-source workflow introducing standardized processing and analysis of RNA and small RNA data. SePIA’s modular design enables robust customization to a given experiment while maintaining overall workflow structure. It is available at http://anduril.org/sepia.

**Electronic supplementary material:**

The online version of this article (doi:10.1186/s13040-016-0099-z) contains supplementary material, which is available to authorized users.

## Background

Massively parallel cDNA sequencing technology (RNA-seq) generates vast amounts of biological data and has become a primary means of transcriptome analysis. A number of RNA-seq protocols exist to produce total RNA, poly(A)-derived RNA and small RNA data. The choice of RNA-seq protocol affects the design principles implemented in the computational analysis of the data [1]. Accordingly, strategies have been developed to computationally identify and interpret biological information from different RNA-seq data types [2–4]. However, these strategies are generally limited to a single data type or integration alone, with a set number of tools and little to no support for extensibility. Furthermore, the accuracy of existing tools often varies across datasets and data types, making a consensus on best practices for processing and analysing sequenced data challenging to reach. A solution to this issue is a modular computational platform that allows testing, development, and easy replacement of methods.

Here, we present the open-source workflow for automated RNA-seq processing, integration and analysis (SePIA). It provides essential pipeline infrastructure for efficient and reproducible analysis of total RNA, poly(A)-derived RNA, small RNA, and integrated microRNA (miRNA) and mRNA data. It does so by (1) expanding the utility of the pipeline engine Anduril [5] to include a bundle of RNA-seq tools and (2) by providing users with the framework in which to best utilize the tools: a set of executable but highly configurable pipeline scripts that, together, form one compendious workflow.

Currently, over 20 existing software and packages (Table 1) are incorporated in SePIA. These tools represent commonly known and established methods developed for sequencing data. Components within pipelines provide users the option to choose amongst functionally similar tools, while built-in transitional steps standardize the input and output formats of these tools for seamless data flow. Through these components, processes are standardized and overall workflow structure is maintained. While the choice of tools is solely up to the user and the needs of their data, the workflow offers the convenience of ready implementation.

**Table 1:** Command-line executable software readily implemented in the SePIA workflow and used with the case studies

Module	Component	Software	Reference
Preprocessing	Adaptor and quality trimming	FastX-Toolkit	hannonlab.cshl.edu/ fastx_toolkit/
		Trimmomatic	[62]
		Trim Galore	www.bioinformatics. babraham.ac.uk/ projects/trim_galore/
	Quality statistics	FastQC ^*a*^	www.bioinformatics. babraham.ac.uk/ projects/fastqc
Read mapping	Align sequences to a reference	BWA	[63]
		Tophat	[64]
		Bowtie	[13]
		Bowtie2	[65]
		STAR	[7]
	Alignment sorting and conversion	SAMtools ^*a*^	[66]
		Picard tools ^*a*^	broadinstitute.github. io/picard
	Alignment statistics	RNA-SeQC	[67]
		RSeQC	[68]
Expression	Mapped reads quantification	HTSeq	[69]
		Cufflinks	[70]
Analysis	Variant calling	Bambino	[35]
	and annotation	ANNOVAR	[71]
	Differential expression	Cuffdiff	[18]
	R bioconductor packages for differential expression	DESeq	[15]
		DESeq2	[72]
		DEXseq	[73]
		EdgeR	[14]
	novel miRNA discovery	miRanalyzer	[74]
		miRDeep2	[75]
miRNA-mRNA integration	R package for SQLite query	sqldf ^*a*^	[76]
	Pathway impact analysis	SPIA	[77]

Software marked with ^*a*^ are mandatory requirements for the minimum execution of a module. A list of software pre-installed within SePIA’s Docker image and the full range of currently available components can be accessed through the website

Included in the workflow are two novel processes for improved integrated analysis of miRNA and mRNA datasets. The first is a database creation pipeline able to integrate and query multiple, user-downloaded public resources (e.g. of predicted and experimentally validated mRNA targets of miRNAs) or custom lists of regulatory pairs. The second is a pair of target prediction components utilizing sequence complementarity to discover likely targets of putative novel miRNAs and to increase the number of potential mRNA targets of known miRNAs.

The SePIA workflow was designed for straightforward execution of pipelines by users of all programming experience. Defining inputs and parameters, for example, is the minimum requirement by both graphical and command-line RNA-seq pipelines, requires no programming, and is done in a single settings file. Installation of the workflow is also simplified to a few copy-and-paste command-lines on a Linux distribution. However, in order to make changes to the pipeline execution and take advantage of other tools available with the workflow, some programming experience is required. Fortunately for method developers and users willing to do some learning while doing, features implemented with the workflow provide a stable environment for straightforward modification and addition of components in a pipeline. Examples, including a fully executable demo case, installation instructions, component documentation, workflow scripts and resources are available on the website, http://anduril.org/sepia.

## Implementation

SePIA is an open-source workflow designed to meet three general aims: (1) to make available a selection of state-of-the-art RNA-seq tools and methods with minimal effort to run, (2) to automatically organize computational results in reproducible, presentable, and easy-to-use formats for in-depth investigation, and (3) to provide a framework for integrated RNA and small RNA processing and analysis. To meet these aims, SePIA relies on the following features supported by the workflow engine Anduril [5]:

- Abstraction of multiple tools and executable scripts written in different programming languages, sparing the user the task of familiarizing with each language.
- Optimization of available computer resources, including parallelization of independent components.
- Modularity. Tools and resources are implemented as independently executable components made linkable through the standardization of input and output formats.
- Verbose logs of executed processes for directive error tracking.
- Implementation of filters and fail safes, including data type checking, to prevent execution and consequent failure of software with invalid/insufficient input.
- An ability to pick-up processes from a previous state, in case of failure or other interruption, and avoiding unnecessary re-execution of parts of a pipeline.
- Complete and easily searchable documentation of all components and their parameters.

Anduril’s Java-based workflow configuration language allows users to set component resources and parameters, and to connect data between otherwise independent components for seamless data flow. The configuration language makes no contribution to low-level processing, providing a clear separation between workflow development and application development. Anduril also contains programming interfaces that support the implementation of custom components written in any command-line executable program such as Bash, Java, R, Python, Perl and MATLAB. Users can thus create custom scripts for a pipeline in their preferred language.

SePIA is built on Anduril and thus inherits the necessary infrastructure and features for reliable large-scale data analysis. Computational overhead produced by a workflow built on Anduril versus simple Bash scripting was explored by Rantanen et al. [6], with the underlying pipeline engine showing improved processing (and dramatically reduced re-processing) time at the cost of computational space for additional files created. These include log files on a global and component level containing verbose output from the executed process, parameters used, input and output file locations. A folder is generated for each process instance executed and files created are immediately accessible after generation. These additional files push up the computational overhead but facilitate process tracking by the workflow engine, fast re-execution, user error-debugging, and future reproduction of results. A summary of time and disk space usage of components, as well as a list of component instance folders produced, are generated outputs of pipelines to further aid users in finding intermediary files and allocating computational resources.

Though the underlying pipeline engine does not enable parallel computing nor regulate computational resources by default, a user can specify these in SePIA execution scripts with normal and locally-defined command-line switches in the header or as input string parameters to select components (e.g. memory-intensive read-mapping software). An example enabling multiple threads is described with performance statistics in the Discussion.

SePIA runs on Linux and assumes all software and dependencies are discoverable in PATH prior to script execution. While some tools and libraries are installed with Anduril and the bundle of RNA-seq components (e.g. R and Python packages), it was not possible for all tools incorporated for use with the workflow. Manually setting up a Linux machine to run SePIA, especially one with custom settings, can thus be daunting and time-consuming.

To simplify installation and enable immediate use of the SePIA workflow, a Docker image was made of a Ubuntu 14.04 distribution with Anduril and essential sequencing tools pre-installed. Docker (http://www.docker.com) is an open-source implementation of lightweight Linux containers in which an ’image’ of an application and all its dependencies are packaged to run in a standardized way. Local folders can also be mounted to Docker containers, which means SePIA can use all the computational resources within the container but access and write files locally. This includes essential log files for workflow process tracking. Docker thus provides a convenient and static environment for pipelines to be executed in with guaranteed result reproducibility. Installation and set up is reduced from multiple required dependencies to Docker and any software for optional execution scripts (e.g. novel miRNA discovery) that a user may wish to run. A demo is available on the website showing how, with a mere six lines of copy-and-paste code, one can install Docker with SePIA and run a complete small RNA pipeline from pre-processing to differential analysis.

Use of parallel computing and resource management with Anduril and memory-consuming software such as STAR [7], however, may be limited within a Docker container despite available run configurations. For some processes in the workflow, manual installation of Anduril and select software may be more convenient. Availability of disk space is also a common problem with RNA-seq. Both issues are addressed with the modular design of the SePIA workflow, which allows for the pipeline and not the dataset to be broken into more digestible parts. This way, a dataset can be processed as a whole by independently executable, compartmentalized, and connectible parts. The entire RNA pipeline, for example, is divided into three script files with individually specified execution folder locations for easier management. Each subsequent script references the previous script’s output to continue the pipeline.

### Software design

A major feature of SePIA’s design and implementation is that it facilitates rapid methods development and parameter fine-tuning, providing users a convenient means to establish their own standard for processing and analyzing different RNA-seq data. While interactive RNA-seq applications such as wapRNA [4], sRNAbench [8], and Galaxy Browser [9] are attractively accessible as they require little to no prior knowledge of programming or installation of software, they are limited in scalability and adaptability to large and complex experiments. The modular framework of SePIA simplifies the task of implementing changes to workflow processes or execution by making direct configuration of driver scripts possible. The workflow can thus be adjusted to work with the data, rather than the reverse which is required by graphical applications. An example is described in Additional file 1, Case 1. Transitional tools simplify and automate file modification within the workflow to meet rigid data type and format restrictions of third-party software, eliminating the need for manual manipulation between steps.

The workflow is designed to readily incorporate the use of over 20 tools but is not limited to these tools. More can be found in the bundle of RNA-seq components that comes with SePIA. Survival and pathway analysis, for example, are available components not included in the pipeline scripts by default. Programming interfaces can be implemented to pipelines like components to include, for example, executable libraries. Combined with the workflow’s modular design, these provide a straightforward means to incorporate any tool or method to a pipeline.

Primary execution scripts are configured to reference a master settings file for a given pipeline. These text files contain a number of parameter options and inputs that enhance the workflow’s ability to accommodate a range of RNA-seq features, but also emphasizes the importance of users first understanding the features of their data and what they aim to get out of it before choosing parameters. For example, users interested in novel transcripts may choose TopHat or STAR as their aligner because of their transcript discovery mode. To aid users, a brief explanation about when, where and what each parameter is for is given in the settings as a guideline.

**Fig. 1:**
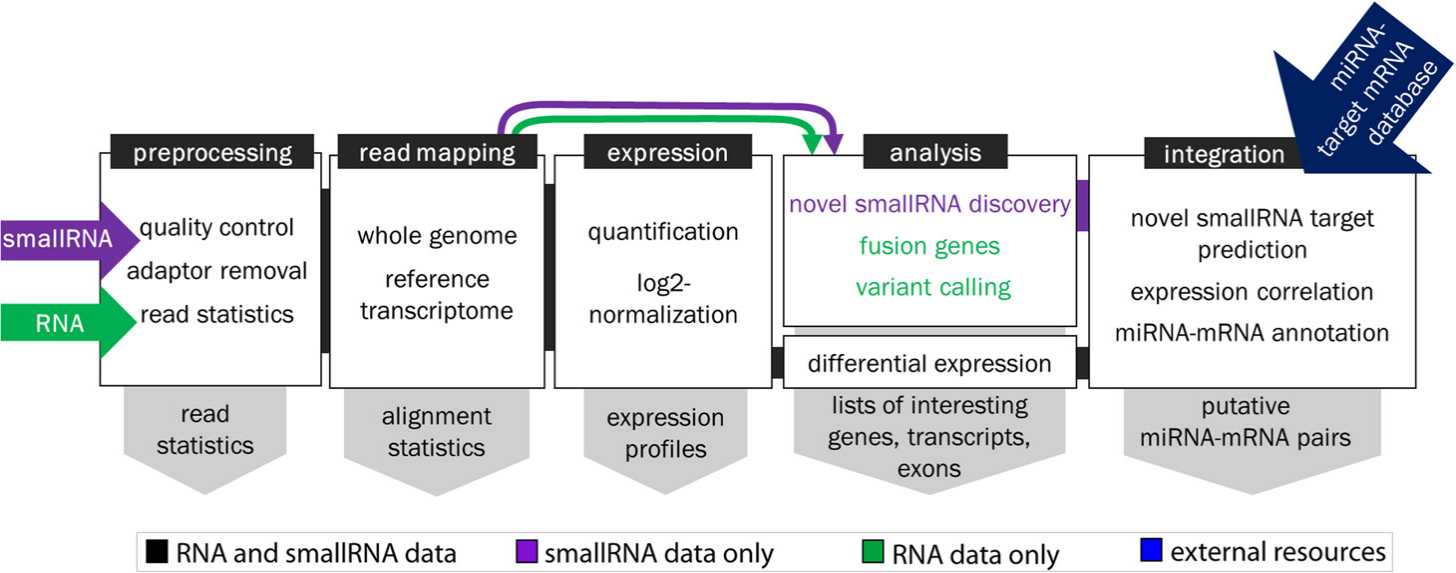
SePIA workflow summarized in five generalized modules. Each module contains a brief description of the major steps performed in each pipeline. For example, the ’double-pass’ alignment means reads are mapped first to the whole genome and then to a reference transcriptome. Colors used represent common processes (black), processes specific to small RNA (purple) and RNA (green) data, and the main outputs of the modules (grey). Incorporation of a miRNA-target mRNA database to the workflow is represented in blue. Interesting molecules of the analysis module are defined as differentially expressed, predicted, or mutated

Components in the workflow are organized into five functional modules representing general steps in an RNA and/or small RNA pipeline (Fig. 1): RNA-seq data preprocessing, read mapping, expression quantification, analysis, and integration. RNA-seq data preprocessing trims adapter and low quality bases from the sequenced reads. Read mapping is done with one of five alignment software and provides methods for estimating parameters such as insert size. Mapped reads are quantified and normalized for gene, transcript, and exon-level expression. Differential expression analysis is performed and, depending on RNA-seq type, optional sequence analysis. This includes novel miRNA and small RNA discovery, variant calling and functional annotation, and gene fusion detection. Further integrated analysis of miRNAs and target mRNAs incorporates third-party target database resources, built-in target prediction based on seed-complementarity, and pathway analysis.

Summary statistics and result files from each module are collected in the *output* folder of a pipeline’s execution path. Contents of this folder are refreshed on each execution of a pipeline. These outputs are then retrieved across multiple execution paths with the SePIA reporting scripts and organized into easy-to-browse HTML reports. Further detail on these reports are provided in Additional file 1.

Modules in SePIA are executed sequentially but it is also possible to execute them independently, allowing users to import previously processed or analyzed data (e.g. already trimmed reads, quantified expression matrices, predefined lists of interesting genes) for further investigation. SePIA is structured to allow for the execution of components as soon as resources and input becomes available (e.g. expression quantification of a sample starts once the alignment file is ready even though other samples are still being aligned), and components that require multiple inputs to be processed (e.g. differential expression analysis) will wait to execute when all inputs are ready. This prevents downstream analysis with incomplete data, and ensures each component produces valid results in a module.

### Preprocessing RNA and small RNA

The mandatory input files for SePIA’s first module are tab-delimited text files containing the following information: unique, per-sample IDs and the corresponding path to unprocessed.fq/.fastq file. Two optional columns can be added to provide further identification of ‘treatment’ and ‘sample’ information, which are required for differential analysis and visualization. Example inputs are provided in Additional file 2.

Three adapter and quality trimmers are implemented in SePIA to cover different features in processing RNA and small RNA sequences [10]. To determine and verify optimal trimming parameters, quality checks are first performed on raw fastq files by FastQC. Read statistics, adapter trimming, and quality control are then done for each input file in parallel. SePIA parameters include two user-defined filters to identify and exclude samples with poor quality scores or with insufficient number of reads surviving from the adapter and quality trimming.

**Fig. 2:**
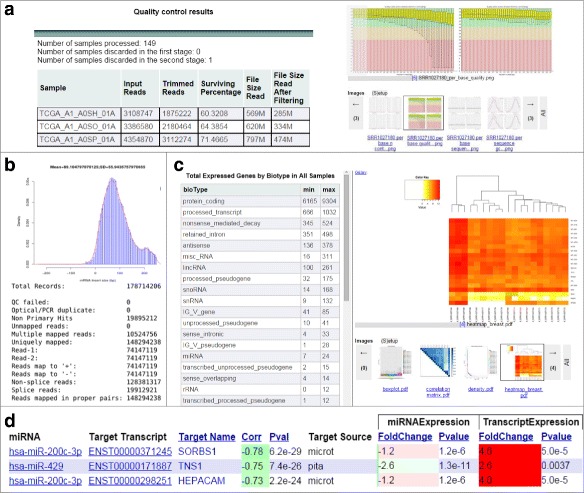
A snapshot of the reports created by SePIA for the case studies. **a** Small RNA preprocessing report for Case II, including FastQC results organized by patient sample. **b**, **c** Alignment and expression statistics for Case I with some standard visualization. **d** The searchable miRNA-target mRNA report for Case II

The output of this module includes an HTML report summarizing preprocessing statistic, FastQC results organized by patient sample or metric type (Fig. 2a), and an array of samples with high-quality processed reads– the primary input for the next module.

### Read mapping and expression quantification

SePIA is equipped to use any of the five available sequence alignment tools listed in Table 1, though using novel tools is possible and straightforward to implement. For read mapping, discovery of potentially novel transcripts, and quantification of alternative splicing, a ‘double-pass’ implementation of STAR aligner is used [7, 11]. A ‘double-pass’ alignment is also used for small RNA data using Bowtie [12, 13] to extract a subset of reads that do not map to existing miRNA annotations for independent novel miRNA and other small RNA discovery.

Mapped reads from RNA data are then quantified for expression at a gene, transcript, and/or exon level using HTSeq and Cufflinks; and for small RNA data at a mature or transcript miRNA level using HTSeq. While Cufflinks produces scaled expression in the form of fragments per kilobase per million (FPKM), HTSeq produces ‘raw counts’ that generally require further normalization to be informative. Three methods are implemented in SePIA for this purpose: counts-per-million with edgeR [14], library-size factor scaling with DESeq [15], or upper-quartile normalization. While the former two represent standard scaling approaches, the latter is a robust alternative for more complex datasets [16].

Small RNA annotations can be extracted from Ensembl general transfer format (.gtf) files and referenced for read quantification. Alternatively, miRNA annotation files in general feature format (.gff3) can be directly downloaded from the miRBase website (http://www.mirbase.org). Though they share a lot of the same genomic information, differences in miRBase’s annotation format compared to Ensembl makes it incompatible for use with HTSeq for expression quantification. SePIA solves the problem with a Boolean settings parameter *mirbase_gff* that, when true, calls a transitional step to reformat the.gff3 file to resemble that of an Ensembl.gtf file. This enables expression quantification on a mature miRNA level not possible with Ensembl transcript annotation.

Outputs from this module include BAM alignment files, a report with alignment statistics (Fig. 2b and Additional file 1: Table S7), ‘raw count’ as well as normalized expression in basic and log2-transformed FPKMs on multiple genomic levels, and expression statistics (Fig. 2c). Both STAR and TopHat can be configured to detect fusion genes and provide the chimeric reads as additional output.

### Sequence and differential expression analysis

The analysis module of the SePIA workflow can be configured with different goals for each dataset. Given that RNA-seq provides base level resolution, variant calling can be performed and functionally annotated to find mutations in expressed genes that may play a role in a disease [17]. Differential expression analysis can be performed with one of several Bioconductor R packages or Cuffdiff [18].

Demonstrating the ease in which SePIA can produce comparable results from different methods, multiple differential expression analyses can be executed in parallel by a single RNA-seq component (DEan). If a user defines a complex dataset with more than two groups in the sample and treatment information, differential analysis will be performed on all paired combinations of treatment groups. These are particularly useful for bioinformatics developers, who are often required to gain results from multiple approaches or with multiple parameter values for comparison.

Novel miRNA and other small RNA discovery is performed on the subset of sequenced small RNA mapping to the genome. MiRanalyzer and miRDeep2 are currently implemented in the workflow. The former features detection of low-abundance candidates and the latter’s conserved approach reduces false positives [19]. Counts of reads mapping to putative miRNAs are produced for each patient sample and merged by SePIA into overlapping genomic regions. This broadens novel miRNA discovery to include genomic regions that may contain other small RNAs encoding miRNAs or having putative sequence features similar to miRNAs, such as small-nucleolar RNAs (snoRNAs) [20, 21]. The result is a single count matrix of sequence regions with putative miRNA-like function. Components for normalization and differential expression analysis are then easily implemented with this count matrix as input. Further annotation and hierarchical clustering of differentially expressed, putative novel miRNA regions by SePIA provides relative genomic context and helps identify potentially interesting regions.

### Integrated miRNA-mRNA analysis

To date, miRNAs have the most consistent and well-characterized regulatory function of all known small RNAs. To evaluate this function for differentially expressed miRNAs, however, their mRNA targets must first be identified. One approach that produces encouraging results is to integrate known miRNA- and mRNA-level expression data with in silico target predictions to filter subsets of potentially interesting miRNAs and their target genes in a particular experiment [22–24]. In SePIA, correlation is used to identify mRNA expression profiles showing a significant, inverse relationship to differentially expressed miRNAs.

For target prediction, SePIA has an optional pipeline, MirTPdb, to create, query, and maintain an integrated SQLite database of predicted and validated miRNA-mRNA pairs from public resources. MirTPdb consists of two components: one to create the SQLite database, and one to query it. The database produced for our case study is comprised of predicted miRNA-mRNA pairs downloaded from TargetScan [25], MicroCosm [26], DIANA-microT-CDS [27], and PITA [28]. It also includes experimentally validated miRNA-mRNA pairs downloaded from release 4.5 of miRTarBase [29] further curated by us, and is available for download on the SePIA website. Multiple target prediction databases were chosen to gain sufficient representation of empirically derived ‘rules’ by which miRNAs are considered to function in the prediction algorithms. The most prevalent of these rules searches for a degree of sequence complementarity between a miRNA’s seed region of 2-7nt, and the 3’UTR of a target mRNA.

To identify targets of predicted novel miRNA regions or to expand the set of targets for known miRNAs, target prediction based on miRNA sequence complementarity was developed for SePIA’s integration module. All known and novel miRNA-mRNA pairs with highly anti-correlated expression profiles and support for their functional interaction from target databases or sequence complementarity analysis are reported in an easy-to-browse website (Fig. 2d). Users can view all mRNA targets of a single miRNA (or vice versa) and browse results by correlation coefficient, supporting evidence for regulation, or expression fold change. A full example of this report is available at http://anduril.org/sepia.

Integrated analysis of miRNAs and mRNAs is a dynamically developing field of RNA-seq research. While correlation is the most common approach incorporated to analyses identifying likely miRNA targets, it is not the only approach. Demonstrating how the workflow’s modular pipeline architecture supports customized application and methods development, we also include an alternative Bayesian approach to identifying functional miRNA and mRNA relationships with GenMir++ [30] as an optional extension of the integration module. It is briefly described in the Discussion.

## Results

We demonstrate SePIA’s broad utility with three publicly available Illumina RNA-seq datasets consisting of paired-end total RNA, poly(A) RNA, and single-end small RNA. The first dataset comprised of total RNA-extracted mRNA from 17 tumor and 3 normal breast samples, sequenced with Illumina HiSeq, from the Gene Expression Omnibus (GSE52194, [31]). The second and third datasets comprised of Level 1 data of 144 poly(A)-extracted mRNA samples (129 tumor, 15 normal breast tissue) and 149 miRNA samples (133 tumor, 16 normal breast tissue) downloaded from The Cancer Genome Atlas consortium [32]. Per-sample details are summarized in Additional file 2.

We organize the datasets into two case studies: the first to showcase SePIA’s utility for transcript-level sequence analysis; the second to demonstrate integration of mRNA and small RNA data.

### Case I: Breast cancer analysis of mRNA extracted from totalRNA

For this RNA-seq dataset we performed preprocessing, read mapping, expression quantification, and differential expression analysis on a gene and transcript level. One tumor sample (SRR1027187) was discarded during preprocessing because less than 60 % of the reads survived quality and adapter trimming. Successfully trimmed reads were mapped to the human genome NCBI38v76 using STAR aligner. A detailed summary of sequence and expression analysis results, as well as parameters used are provided in Additional file 1. Briefly, the majority of genes found to be differentially expressed between normal and tumor breast tissue were protein coding, with variable detection of small non-coding genes by Cuffdiff and DESeq2. 23 genes were found to have preferential usage of one isoform over others in normal versus tumor samples (Additional file 1: Table S2) and include breast cancer-associated genes *EIF3L* and *EEF2* [33, 34]. DEXSeq identified 546 genes with at least one case of differential exon usage, with a minimum log2-fold change of two.

Variant calling was implemented using Bambino [35] and filtering steps according to Piskol et al. [17] and GATK are implemented in SePIA to minimize the number of false positive calls. We found a total of 85,664 indels and 16,882 SNVs. From these, 2,534 are known post-translational editing sites. When comparing variants found in tumor against normal samples, 341 variants were overrepresented in tumor tissue comprising 246 genes. The list of the variants found in exonic regions in protein coding genes is included in Additional file 1: Table S4.

### Case II: Integrated breast cancer analysis of miRNA and poly(A) derived mRNA datasets

From the 144 mRNA samples, 138 passed quality control with one sample discarded for having mostly poor quality sequences (i.e. an average quality score less than 20), and five samples discarded for having less than 60 % of reads surviving trimming, an indicator of poor sequence content. Of the TCGA miRNA dataset, 148 of 149 samples passed quality control, with one sample discarded for having less than 20 % of reads surviving trimming. The low percentage indicates either poor sequence content after trimming or poor miRNA representation in the sample.

Preprocessing, read mapping, and expression quantification of poly(A)-derived mRNA data was performed with the same tools as in Case I. 4,170 genes and 2,257 mRNA transcripts were found to be differentially expressed between normal and tumor breast tissue with a conservative false-discovery rate of 0.05. Furthermore, 48 genes had preferential usage of one isoform over others in normal versus tumor samples (Additional file 1: Table S3).

MiRNA sequences were aligned with Bowtie to known miRNA transcripts (miRBase, version 21). On average, 95 % of our preprocessed small RNA map to the genome, with approximately 1–33 % mapping to something other than known miRNAs in the genome. Eipper–Mains et al. [36] suggests some of these reads map to other small RNAs, which concurs with our rationale to extend novel miRNA discovery to include other small RNAs.

Though SePIA can quantify miRNA expression on a mature and precursor level, only mature miRNAs were calculated for this study. Lengths of trimmed reads were normally distributed around 22nt, indicating content consistent with mature miRNA sequences. The human annotation file hsa.gff3 was retrieved directly from miRBase to create the reference for HTSeq quantification. 28 % (799/2,813) of all human mature miRNAs were expressed in the normal and tumor breast tissue samples with at least two mapped reads, consistent with tissue-specific expression characteristic of miRNAs [37].

Demonstrating the ease in which SePIA can produce comparable results from different approaches, three methods were used for differential expression analysis: DESeq, EdgeR, and upper-quartile normalized t-test. Bioconductor package edgeR [14] identified the most differentially expressed mature miRNAs (*n*=408 with false discovery rate <0.05) shared by all three methods.

Novel miRNA and other small RNA discovery utilizing miRanalyzer produced a matrix of 442 putative novel miRNA regions between 72 and 159nt in length. In comparison, the range of known miRNA transcripts in miRBase v.21 is between 40 and 179nt. Analysis identified 33 of these putative novel miRNA regions to be differentially expressed between normal and tumor breast tissue (false-discovery rate <0.05). Encouragingly, manual inspection of these novel regions revealed five overlapping snoRNAs (*SNORA56, SNORA69, SNORD95, SNORD49, SNORD82*), one overlapping a predicted novel miRNA (*AL161626.1*), one overlapping a known miRNA not in miRBase (*FP236383.10*), and one small zinc-finger protein (*ZNF813*). SnoRNAs that encode miRNAs or have miRNA-like function have been previously reported [20, 21]. Independent alignment of small RNA sequences to small non-coding RNA transcripts with Ensembl IDs (size-selected for 15–80nt) revealed an additional 1.3 % of total processed reads mapped to small RNAs other than miRNAs, particularly snoRNAs with a C/D box and transfer RNAs. Similar percentages were reported by Eipper–Mains et al. [36].

In this study, we implement the integration module to further evaluate 408 differentially expressed miRNAs (plus 33 differentially expressed putative miRNA regions) and their roles in breast cancer. Integration was performed on 123 tumor and 15 normal samples sharing patient sample IDs in both the TCGA miRNA and mRNA datasets. Target transcripts of the differentially expressed miRNAs were identified with our target prediction database, with anti-correlated expression (Pearson coefficient <-0.4 and false discovery rate <0.01) [38, 39] and with a minimum absolute log2 fold-change of 0.5. A total of 4,208 pairings between 174 differentially expressed miRNAs and 915 transcripts with anti-correlated expression and supporting annotation. We used SePIA’s sequence complementarity components to increase the total number of putative targets for our differentially expressed miRNAs and gained an additional 8036 pairings containing 217 miRNAs and 1809 gene transcripts not recorded in our integrated target database.

We found 256 differentially expressed transcripts predicted by SePIA’s sequence complementarity components to have binding motifs for our 33 putative novel miRNAs and anti-correlated expression (Pearson coefficient<-0.4 and false discovery rate <0.01). Stringent binding motifs (8mer, 7mer-m8, 7mer-a1) were found in 115 of the 373 predicted miRNA-mRNA pairs. These include two down-regulated tumor suppressor genes *CCNDBP1* [40] and *AHNAK* [41] targeted by a putative miRNA region in chr2:17753239-17753361 (-), and two overexpressed genes *MYO10* [42] and *CCNB2* [43] targeted by a putative miRNA region in chr5:181243270-181243420 (-), which overlaps *SNORD95*. These genes are known to play essential roles in breast cancer tumorigenesis, invasion and metastasis [40–43].

#### The mir-17/92 cluster of oncogenes

Amongst our list of differentially expressed miRNAs are members of the mir-17/92 cluster, the most frequently activated miRNA cluster in cancers. All six members (miR-17, miR-18a, miR-19a, miR-20a, miR-19b and miR-92a) are located within the third intron of the *C13orf25* gene at 13q31.3, and are well-known promoters of cell proliferation, cell survival, and angiogenesis [44]. Indeed, these miRNAs were significantly overexpressed in our case study’s breast tumor tissues. This highly-conserved sequence cluster also has two human paralogs whose members were also overexpressed in breast tumors (specifically, miR-106a, miR106b, miR-93, miR-20b, miR-92a-2 and miR-363) and support complementary roles to the mir-17/92 cluster in tumorigenesis [45].

**Fig. 3:**
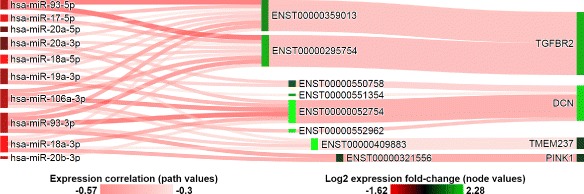
Target genes of the mir-17/92 cluster and paralog clusters in the TGF-beta signaling KEGG pathway. Target transcripts were selected to have minimum log2-fold change of 0.5 between tumor and normal breast tissue. Node colors represent expression fold change between normal and tumor breast tissue samples. Relationships between connected miRNA-target transcript pairs are shaded based on correlation coefficient values. Connections between transcript and gene represent average correlation values of contributing transcripts and their regulating miRNAs

Three commonly predicted targets of the miR-17/92 cluster showing anti-correlated expression are tumor suppressors *TGFBR2* [46], *DCN* [47], and *CAV1* [48] which were under-expressed in the breast tumor tissues. This is consistent with previous studies presenting the mir-17/92 cluster as inhibitors of TGF-beta signaling via regulation of key pathway genes [46]. Anti-correlated and predicted miRNA-target gene pairs of the TGF-beta signaling pathway and mir-17/92 cluster are illustrated in Fig. 3. Interestingly, SePIA’s integrated analysis reveals putative differences in miRNA co-regulation of *TGFBR2* and *DCN*: 9 of the 10 expressed members appear to target both of the known transcripts of *TGFBR2*, the ligand-binding receptor for all members of the TGF-beta family, while all 10 miRNAs appear to co-regulate four different transcripts of *DCN*, an anti-angiogenic factor in breast cancer [47]. Similarly, members of the mir-17/92 cluster and paralog clusters were predicted by our sequence complementarity components to co-regulate three different transcripts of *CAV1*, whose role in breast cancer has been extensively studied [48, 49] but had not been previously linked to miR-17/92.

## Discussion

The SePIA workflow allows analysis of paired-end total RNA, poly(A)-derived RNA, and single-end small RNA data. All three data types were processed by the same RNA-seq components at common steps in the workflow, such as preprocessing and alignment, to produce standardized outputs. The ease in which SePIA’s pipelines can be extended was demonstrated in Case I, where variant calling was performed as an additional, independently-executed analysis of transcript-level data. Integrated analysis was demonstrated in Case II, identifying biologically interesting miRNA-mRNA pairs by combining expression correlation with in silico target predictions [24]. Though Cufflinks and HTSeq are used in the workflow for mapped read quantification, users may prefer other tools such as RSEM [50], eXpress [51], and BitSeq [52]. These are available as components that can also be used with SePIA and instructions on how to implement them is given on the SePIA website. Users with a basic proficiency in a programming language are thus able to utilize the workflow’s modular framework to implement and test custom components, replace processes, or implement independent approaches to run in parallel.

All workflow scripts have a ’.and’ extension and are written in Anduril’s configuration language based on Java. Users of any member of the Java programming language and comparable languages like Python will, therefore, find it relatively straightforward to read the pipeline execution scripts and learn how to further configure them.

SePIA’s sequence complementarity components discovered potential target mRNAs for small RNAs with miRNA-like features, providing association to biological function. It was also used to increase the number of predicted targets of differentially expressed known miRNAs. Sequence complementarity is widely accepted as a mechanism in which miRNAs identify targets. In Case II, 174 of the 408 differentially expressed miRNAs had targets from our integrated target database with anti-correlated expression and minimum fold-change of 0.5. Sequence complementarity added 54 miRNAs with potential binding motifs in the 3’UTR regions of their anti-correlated targets. The number includes tumor suppressive (mir-205 and mir-143) and oncogenic miRNAs (mir-187 and mir-27b) reported in breast cancer [53–56]. All 102 putative miRNA-target pairs from this additional set contained stringent binding motifs of 8mer, 7mer-m8 and 7mer-a1, presenting this approach as an opportunity to explore viable cancer-related miRNA regulation events which have not yet been previously reported.

Correlation is an intuitive and straightforward approach to integrating miRNA and mRNA expression. Putative miRNA targets supported by anti-correlated expression is valuable information for planning any experimental work with miRNAs. However, to identify functionally interesting networks of miRNAs and mRNAs from expression data, users may prefer more complex integrative approaches such as GenMiR++ [30]. An alternative and independently executable extension of the integration module demonstrates how SePIA can streamline the preparation of necessary input files and execution of the software. The output of the pipeline is a tab-delimited text file of miRNA-mRNA pairs with GenMiR++ scores that can easily be used with further downstream analysis. The file is included in the results reported on the SePIA website.

Tools available with SePIA are generally free to use. Most dependent software and packages are also included with the Docker installation of SePIA. However, while it is possible to run MATLAB scripts with the workflow, MathWorks license prevents us from including the software. Therefore, to implement a custom MATLAB script in a pipeline or to use the optional integration pipeline with GenMiR++, users must first install the software themselves.

SePIA pipeline scripts have been set up for the general process and analysis of a two-case RNA-seq dataset. Users with such a dataset need only to change the inputs and parameters outlined in the master settings file to run the pipeline. A dataset with more than two cases will, however, affect how differential analysis is performed and pipeline modification is required. The amount of modification to be done ultimately depends on the programming experience of the user and willingness to troubleshoot a pipeline script. A user can, at the very least, disable components in the analysis module incompatible with their dataset by simply commenting them out. Similarly, a user who wishes the pipeline to automatically output a ‘result’ file from a ‘componentA’ rather than retrieve the file manually from the execution folder must be able to add to the end of the pipeline’s execution script the line ‘OUTPUT(componentA.result)’. The pipeline’s modular design thus allows for both component execution and exclusion of independent parts.

The underlying infrastructure of SePIA is designed to be scalable to large computational resources dedicated to high-throughput sequencing analysis, and for optimal use with batch processing. SePIA is capable of automating a time-memory trade-off by running in parallel independent processes and allocating necessary memory or thread requirements for each individual component. However, for some components the time-memory complexity depends on the underlying implementation. To demonstrate, a subset of 8 samples (4 normal, 4 tumor) was extracted from the TCGA miRNA dataset to run on an Intel Xeon X5650 processor with 90GB RAM and a 24-core. Using eight computational threads, preprocessing, alignment, quantification, and differential expression analysis was performed in less than 70 minutes. Novel miRNA discovery was fast with an average 40 minutes per sample, but the single act of annotating putative novel miRNA regions with information on their relative genomic location was time-intensive and required an additional 24 hours to perform. Performance statistics is available in Additional file 1.

High-throughput sequencing of RNA is powerful but it is not without its challenges [57]. With inevitable advancements in RNA-seq technology and in non-coding RNA research, no workflow can be all-encompassing without an ability to evolve. The available tools and modular design of SePIA is thus essential for implementation of novel RNA-seq data processing and analysis tools to the workflow. For example, cross-linking technologies such as CLASH [58] make it possible to identify direct RNA-RNA hybridization. The consequent influx of available experimentally validated interactions could be easily included in the MirTPdb target database.

With changes in RNA-seq technology and research comes changes in softwares and algorithms used in pipelines. Often, these changes can break pipelines and prevent reproducibility of results. Implementation of SePIA with a Docker container ensures a reliable and static environment with all the required tools in compatible software versions is always available to run SePIA. Furthermore, we will update the Docker container with new releases of the SePIA workflow to ensure continued usability.

Though users can install and update software manually, there is no guarantee components using the software will continue to work correctly. Troubleshooting components requires some programming skills and because SePIA is built on Anduril, online support is available to the user. The bundle of RNA-seq components uses versioning and it is possible to update to development versions of the RNA-seq components.

Like RNA-seq, the SePIA workflow continues to be developed. Future releases will provide ready execution of additional tools, like the previously mentioned RSEM, as new RNA-seq components. Version updates of STAR, Picard, and other listed tools may alter component implementation in the workflow. Currently, version information is printed to log files by most components available for use with SePIA but not by all. This will be resolved in a future release to further ensure reproducibility of results. Fusion gene detection will be a standardized process supporting the use of ChimeraScan [59], FusionCatcher [60], and deFuse [61]. Novel and existing components will be developed to support single-cell RNA data. Finally, methods to analyze both long and small non-coding RNAs will be further explored.

## Conclusion

We developed SePIA to address the need for a high-throughput workflow that provides a standard framework to process and analyze multiple RNA-seq data types, primarily small (<200nt) and long (>200nt) RNA. The built-in pipeline infrastructure, modular design, and range of independently executable components of the workflow supports advanced methods-development and benefits users who regularly process RNA-seq data. Pipeline scripts can be minimally configured with basic programming knowledge. For novice users, the master settings file and ready-to-use computational environment in Docker makes it easy to input data and run pipelines with minimal effort.

Pipeline extensibility is a key advantage of the modular framework. It supports implementation of alternative computational approaches and the development of further downstream analysis. To assist with functional studies of regulatory small RNAs, SePIA features tools to query and maintain a SQLite database of annotated interactions, and to discover putative novel miRNA-mRNA interactions.

The versatile design of the SePIA workflow makes it straightforward to incorporate developments in RNA-seq technology and customized applications. Components for use with the workflow are constantly being developed for further user convenience. SePIA thus achieves a ’middle-ground’ approach to RNA-seq processing and analysis that harnesses the flexibility and computational resource management of bash scripting with the approachable usability and ease of one-command pipeline execution of more graphical user interfaces.

## Additional files

Additional file 1 Supplementary information. PDF containing supplementary methods and tables, pathway impact analysis, heatmap of differentially expressed miRNAs and mRNAs per case study, SePIA pipeline outputs and performance statistics. (PDF 1176 kb)

Additional file 2 SePIA workflow scripts and files. A zip file containing essential workflow scripts and files, including full sample information for the case study. Scripts are configured to run in Docker and to reference a mount point called *SePIA_external*. See the README for instructions. (RAR 235 kb)
