# Supplementary material for: SePIA: RNA and small RNA sequence processing, integration, and analysis
Source: BioData Min. 2016 May 20;9:20. doi: 10.1186/s13040-016-0099-z (PMC4875694; doi:10.1186/s13040-016-0099-z)
Supplement: Additional file 1 — Supplementary information. PDF containing supplementary methods and tables, pathway impact analysis, heatmap of differentially expressed miRNAs and mRNAs per case study, SePIA pipeline outputs and performance statistics. (PDF 1176 kb) [file 13040_2016_99_MOESM1_ESM.pdf]

Contents

Supplementary Methods 1
Case I: Breast cancer analysis of mRNA extracted from totalRNA 1
Case II: Integrated breast cancer analysis of miRNA and poly(A) derived mRNA datasets 2
Pathway impact analysis 3
Performance statistics 3
Pipeline outputs 4
References 7
Supplementary Tables 7
Table 1. KEGG pathways enriched with targets of differentially expressed miRNAs with FDR < 0.05. 7
Table 2. Genes in Case I with splice variants having log2 fold changes in opposite directions in normal and tumor samples. 7
Table 3. Genes in Case II with splice variants having log2 fold changes in opposite directions in normal and tumor samples. 8
Table 4. Exon variants with a higher ratio in normal or tumor samples. 11
Table 5. KEGG pathways enriched with differentially expressed genes from Case I and with FDR < 0.05. 12
Table 6. KEGG pathways enriched with differentially expressed genes from Case II and with FDR < 0.05. 13
Table 7. Alignment statistics of the small RNA read mapping module. 14

Supplementary Methods

Case I: Breast cancer analysis of mRNA extracted from totalRNA

Filters were implemented in the quality control and in the sequence trimming steps of the preprocessing module to uphold user-defined quality levels, identifying and extracting subpar and/or unexpected features of sequencing data prior to analysis. For this study, samples needed a minimum 70% of all pre-processed reads to have an average phred score of 20 or higher across read lengths to pass the first quality control, which all samples did. Trimmomatic was used for quality trimming: first 13 bases were cropped due to uneven per base sequence content as well as leading and trailing bases below phred score 30. Resulting sequences shorter than 25bp were discarded. If more than 30% of the number of reads in the sample was removed on the previous step, then the sample was discarded.

Modifying and extending the SePIA workflow is relatively easy and straightforward. In this study, a sample was initially filtered out in the preprocessing module because no reads survived quality trimming. A look at the basic statistics of the SePIA quality report revealed the sample to be encoded with Sanger/Illumina 1.9 while the other samples were encoded with Sanger/Illumina 1.5. By adding transitional components to the preprocessing module of the SePIA workflow, the sample was successfully re-evaluated with the correct encoding and rejoined to the dataset before proceeding to the next module (see Additional File 2, SePIA scripts).

Successfully trimmed reads were then mapped to the human genome NCBI38v76 using STAR aligner, with chimeric (fusion) detection enabled. The first STAR alignment yielded splice junctions used to build a 'spliced genome', or transcriptome index, to reference in the second STAR alignment. Information on unannotated splice junctions crossed by multiple mapped reads was output for further analysis. Mapped reads were then quantified on a gene (HTSeq, Cufflinks), transcript (Cufflinks), and exon (HTSeq) level.

For differential expression analysis between normal and tumor breast tissue, we used both Cuffdiff and DESeq2. Cuffdiff was first tested with default parameters and 1,422 differentially expressed genes (DEGs) were identified. When using a per-condition dispersion model, the number of DEGs increased to 1,817. For DESeq2, the total number of DEGs (p-value <0.05 and adjusted p-value < 0.1) was 24,052. Further filtering of DESeq2-calculated DEGs using average expression (basemean > 10) and absolute log-fold change > 1 yielded 5,778 DEGs. In all cases, the majority of the DEGs are protein coding. Cuffdiff did not report any differentially expressed pseudogenes while in DESeq2 they account for 12% of the DEGs. Also, the percentage of short noncoding genes detected by Cuffdiff is much smaller than DESeq2, but for long noncoding genes the percentage is the same (Figure S1).

On a gene transcript level, Cuffdiff identified 68 differentially expressed transcripts using default testing parameters, and 123 differentially expressed transcripts using a per-condition dispersion model. The detected isoforms usually belong to genes reported as differentially expressed, but it is also possible to identify genes that express a different variant in each condition. 23 genes were found to have preferential usage of one isoform over others in normal versus tumor samples (Supplementary Table 2), and include breast cancer associated genes EIF3L and EEF2 (Figure S2). For exon-level analysis, we identified 546 genes with at least one case of differential exon usage, with a minimum log2-fold change of 2 using DEXSeq**.**

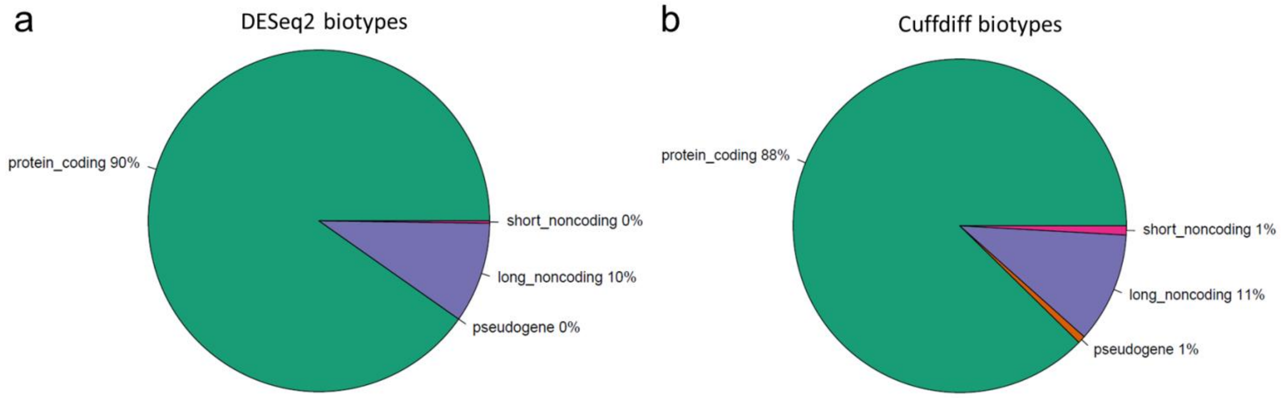

Fig S1. Proportion of DEGs belonging to each class (a) from DESeq2 and (b) from Cuffdiff.

Case II: Integrated breast cancer analysis of miRNA and poly(A) derived mRNA datasets

Based on initial FastQC reports on per base sequence content, the first 10 bases of sequenced reads in the TCGA mRNA dataset were cropped. All other filtering and trimming parameters in FastQC and Trimmomatic were the same as in Case I. Alignment is also as described in Case I, minus the chimeric detection. Expression quantification and differential analysis was performed only on a gene and transcript level with Cuffdiff.

Reads in the TCGA miRNA dataset were trimmed with FastX-Toolkit (http://hannonlab.cshl.edu/fastx_toolkit) and had the following per-sample parameters: sequences were kept if at least 20% of its nucleotides had a base-quality score of 25 or higher, and if it was between 15nt and 32nt in length after adapter removal. A sample was filtered out if less than 70% of the raw sequenced reads survived trimming.

Novel miRNA and other smallRNA discovery was performed on the subset of reads mapping to the genome but not to any known miRNAs using miRanalyzer, which generally produces more novel candidates than miRDeep2 due to the latter's dependence on detecting sufficient read abundance (Williamson *et al.*, 2013). Transitional steps in SePIA merged quantified expression across samples to create an expression matrix of putative novel miRNA regions. Expression was then filtered such that a novel miRNA region had to have at least one mapped read in 10% of the samples analysed.

In this case study, our correlation cutoff of -0.4 corresponds to a false discovery rate < 0.002. In literature, integrated miRNA-mRNA analysis have been performed with a correlation cut-off < -0.3 (Qin *et al.*, 2015) and predicted miRNA-mRNA pairs with expression correlation < -0.4 have been validated (Genovesi *et al.*, 2011; Laxman *et al.*, 2014). Thus, our threshold represents a relatively conservative choice. The miRNA target database created with MiRTPdb was used to further filter out those correlated pairs that were not supported by at least one of the five miRNA-target resources.

In SePIA's integration module, function KnownMirnaTarget retrieves validated and predicted targets of known miRNAs with anti-correlated expression from the integrated target database and from the results of our built-in sequence motif search tool. A similar approach is applied to retrieving targets of novel miRNAs by using the function NovelMirnaTarget, in which binding site motifs (8mer, 7mer-m8, 7mer-a1, 6mer and offset-6mer) from the mature sequence seed regions of the candidate novel miRNAs are searched along the 3'UTR sequences of their anti-correlated target transcripts with the requirement of sequence complementarity. A reporting function MiRNAtargetReport takes the outputs from the two functions and generates a comprehensive HTML report for the integration study.

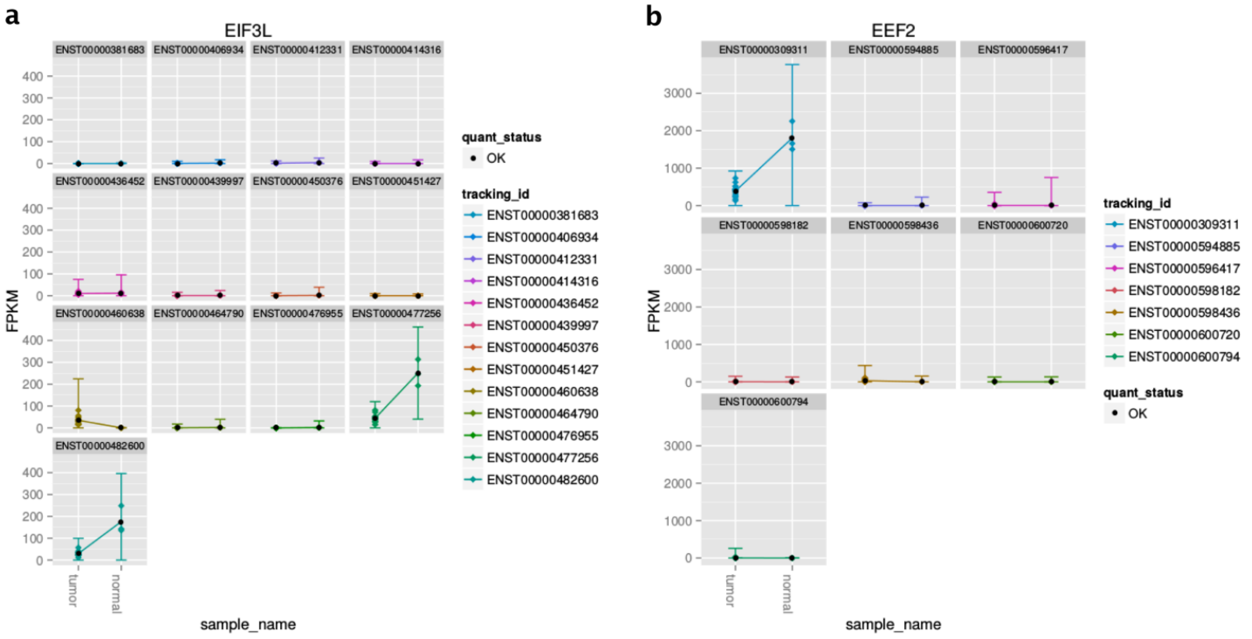

Figure S2. (a) Different isoforms of EIF3L are differentially expressed in tumor and normal tissue: ENST00000460638 and ENST00000477256. (b) Different isoforms of EEF2 are differentially expressed in tumor and normal tissue: ENST00000309311 and ENST00000598436.

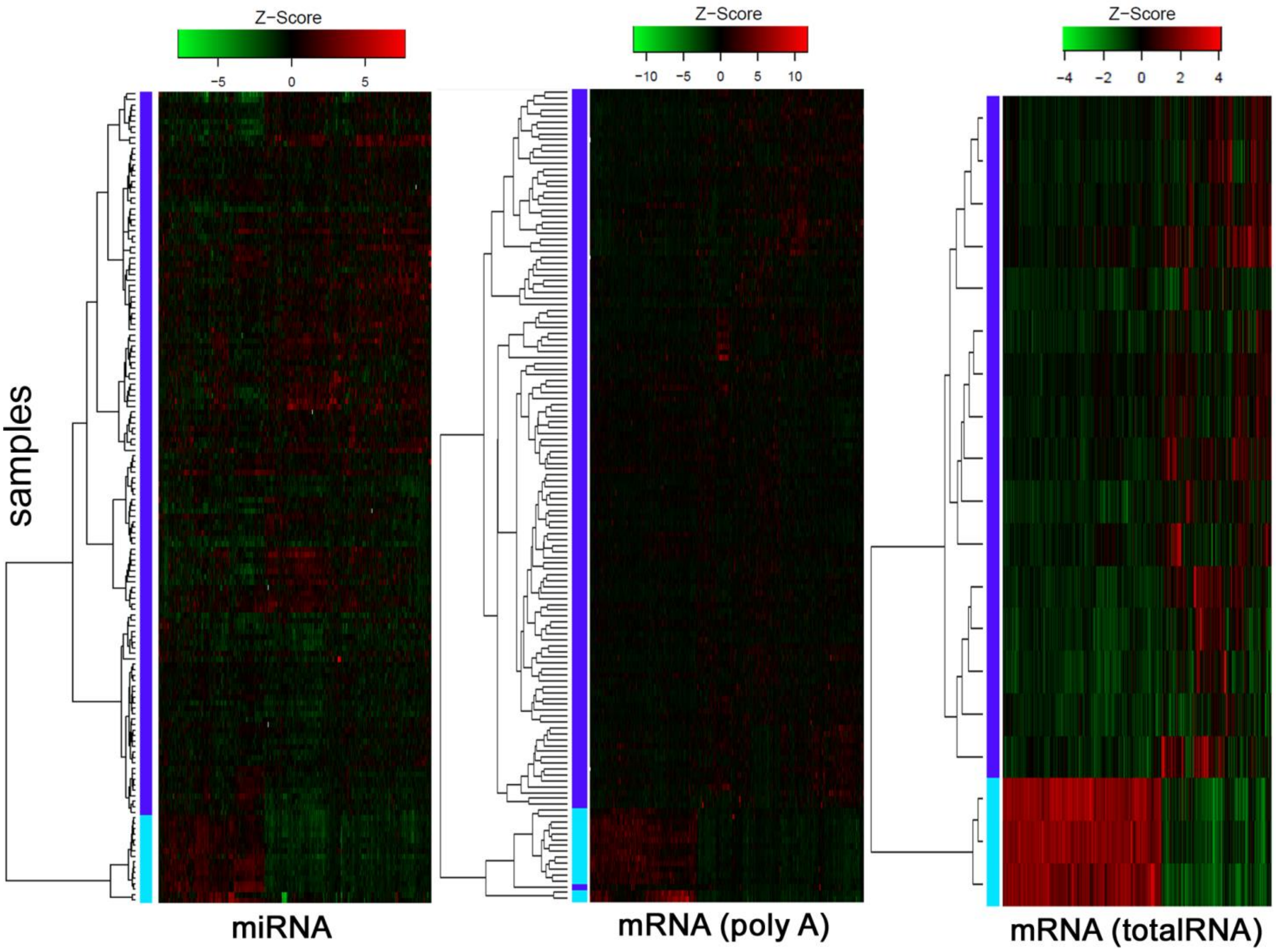

Figure S3. Differentially expressed mature miRNAs (left), transcripts derived from poly(A) RNA (center), and transcripts derived from totalRNA (right). In each case, expression of the normal samples (blue) cluster together and reveal a distinct set of differentially expressed genes for further analysis.

Pathway impact analysis

To discover if our differentially expressed genes contained relevant biological insights to breast cancer, we included pathway impact analysis to the RNA portion of the workflow. Any further downstream analysis would be specific to an experimental design and pushes beyond the scope of this paper. Several components are available in Anduril to facilitate this endeavor and are simple to use in conjunction of SePIA.

To determine possible regulatory roles for the 408 differentially expressed miRNAs in breast cancer, we performed pathway impact analysis on the 3,579 predicted and anti-correlated transcript targets showing significant differential expression between tumor and normal samples and absolute log2-fold change > 0.5. These genes were enriched in many cancer-related pathways (N=33), including the previously mentioned TGF-beta signaling, focal adhesion, cell cycle and the p53 signaling pathway (Supplementary Table 1). This is consistent with current knowledge on their essential roles in breast cancer tumor initiation, proliferation, invasion and metastasis (Luo & Guan, 2010; Bijian *et al.*, 2013; Fernandez *et al.*, 1998; Gasco *et al.*, 2002). Abnormal miRNA regulation of these cancer-associated pathways may reveal essential drivers for targeted cancer therapy.

We also performed pathway impact analysis on all genes found to be differentially expressed (Cuffdiff FDR<0.05 and absolute log2-fold change > 0.5) between breast cancer tumor and normal samples in the TCGA and GEO datasets **(**Supplementary Table 5 & 6**)**. Of the 23 enriched pathways for the TCGA dataset, 14 overlap with pathways enriched with targets of differentially expressed miRNAs and 14 overlap with pathways enriched with DEGs in the GEO dataset. Encouragingly, 8 pathways are significantly enriched in all three gene sets (FDR<0.05) and include focal adhesion, cell cycle, and transcriptional misregulation in cancer. These support an overall contribution of miRNA regulation to breast tumorigenesis.

Performance statistics

SePIA performance was measured on the following subset of 8 miRNA sequenced samples on a single virtual machine running on an Intel Xeon X5650 processor with 90GB RAM and a 24-core.

Subset of TCGA breast cancer smallRNA-seq samples used for SePIA performance.

miRNA sample	Sequence ID	classification
TCGA_E2_A10B_01A	SRR326529	Tumor
TCGA_E2_A10C_01A	SRR326532	Tumor
TCGA_E2_A10E_01A	SRR326510	Tumor
TCGA_E2_A10F_01A	SRR326514	Tumor
TCGA_A7_A0CE_11A	SRR317771	Normal
TCGA_A7_A0D9_11A	SRR317773	Normal
TCGA_A7_A0DB_11A	SRR317760	Normal
TCGA_BH_A0B3_11B	SRR317762	Normal

The miRNA pipeline includes preprocessing, alignment, expression quantification, and differential expression analysis. Novel miRNA and smallRNA discovery is an optional extension of the pipeline and was evaluated separately. Two runs were performed for each version of the pipeline using eight computational threads; one for each sample. The advantage of parallel computing is shown in the difference between cumulative time for all eight samples, and the actual elapsed time of the pipeline. Available computational resources often fluctuate on a virtual machine, and this accounts for differences in maximum, minimum, and median times of component instances between executions.

SePIA performance statistics for the subset of TCGA breast cancer smallRNA-seq samples.

smallRNA	# threads	Cumulative time	Elapsed time	Max/Min/Median time elapsed per component instance	Max memory usage	Total file size
pipeline_01 (no novel analysis)	8	04:37:51	1:07:42	08:36.25/00.08/00:01.18	2.4MB	23GB
pipeline_02 (no novel analysis)	8	05:05:10	1:04:13	33:36:00/00.06/00.88	2.4MB	
pipeline_01	8	1d 13:49:35	1d 04:26:07	24:56:35.00/00.05/00.60	13.8MB	23.2GB
pipeline_02	8	1d 16:14:13	1d 03:52:27	25:00:24.00/00.06/01.23	14.5MB	

From this table, it is clear that novel miRNA and smallRNA discovery contains a single component instance that takes the bulk of execution time. Indeed, we identified this component instance to be the single act of annotating putative novel miRNA regions with information on their relative genomic location (e.g. whether the region is intergenic or intragenic. Identity of the host gene, transcript, and intron/exon).

Pipeline outputs

One tab-delimited output file from SePIA pipelines is *pipeline_statistics.csv*, which gives a general breakdown of all components executed in the pipeline. Total file size processed in a performance run is calculated to include the initial 9.1Gb of raw Fastq input data, which is not copied from its original location but is nonetheless processed as part of the pipeline's execution. Outputs, intermediary files, component documentation, process logs and tracking files generated by the pipeline thus account for the remaining ~13.9Gb. The times recorded below correspond to a separate run from the performance statistics recorded above and emphasize the impact of available computational resources on SePIA’s performance. All component instances executed in a pipeline (e.g. Alignment for each individual sample) are listed in the corresponding output file *pipeline_component_instances.csv*.

Content of SePIA output file *pipeline_statistics.csv*.

Component statistics:

**Name**	**#**	**Sum time**	**N files**	**sizes**
ArrayCombiner	1	0:00:00	2	11.1KB
CSV2Array	1	0:00:00	2	814B
ConfigurationReport	1	00:00:00+	0	0B
DEan	1	0:00:20	5	40.0KB
ExtractCond	1	0:00:00	1	197B
HTSeqExprMatrix	1	0:00:01	7	401.4KB
HTSeqExprNormalize	1	0:00:03	8	667.0KB
LatexCombiner	1	0:00:01	3	12.9KB

LatexPDF	1	0:00:01	5	110.7KB
MDSPlot	1	0:00:01	4	5.2KB
StandardProcess	1	0:00:01	5	114.6KB
HeatMapReport	2	0:00:02	6	21.7KB
LatexTemplate	2	0:00:00	10	73.3KB
ExpressionSubset	3	0:00:00	3	15.6KB
SimpleWebPage	3	0:00:04	29	214.5KB
Array2CSV	6	0:00:31	6	4.5KB
CSVFilter	7	0:00:00	7	14.0KB
Bam2Fastq	8	0:04:19	8	1.8GB
HTSeqCount	8	0:19:05	8	328.4KB
TableQuery	8	0:00:08	8	2.6KB
Folder2Array	9	0:00:00	18	1.2KB
OUTPUT	11	00:00:00+	0	2.2MB
CSVCleaner	12	0:00:00	12	145.2KB
QCParser	16	0:00:05	416	195.5KB
SeqQC	16	0:08:18	160	2.0MB
REvaluate	19	0:00:19	154	723.6KB
ImageGallery	21	0:00:31	325	12.7MB
CSVJoin	25	0:00:08	25	27.4KB
HTMLTable	25	0:00:02	25	2.8MB
Array2Folder	32	0:00:08	159	6.1MB
StringInput	33	0:01:24	33	644B
ArrayConstructor	40	0:00:14	80	36.5KB
INPUT	89	00:00:00+	0	9.1GB
BashEvaluate	98	0:34:45	1694	12.1GB
ArrayExtractor	240	00:00:00+	0	0B

Number of instances: 744
Cumulative running time: 01:10:31h
First to last time: 00:24:15h
Cumulative files produced: 3228
Cumulative file sizes: 23.0GB

Times and file counts are minimum estimates. File counts descend 1 folder level.
File sizes include INPUT component sources.

For user convenience, ***'-report.and'*** execution scripts for each SePIA pipeline retrieves and organizes the main RNA and small RNA outputs by modules into an easy-to-browse HTML report. Useful content descriptions such as individual file sizes are stated in module *index.html* files. Full reports are perusable at http://anduril.org/sepia/download.html#results. The directory tree of the miRNA output folder (generated for performance statistics) is shown next to the corresponding miRNA report output folder in Supplementary Figure 4. The same files are reorganized into modules and given descriptive HTML links. Pipeline performance files, for example, are renamed to be more informative.

The SePIA pipeline for poly(A)-derived RNA and total RNA have the same modular structure with different parameter configurations. Outputs are therefore similarly organized but contain different files. The differences in Supplementary Figure 5 account for (1) separate quality control of total RNA sample SRR1027172 due to a difference in quality encoding, and (2) additional variant calling performed on total RNA samples.

```
mirna_demo_output
-- aligned-pass.csv
-- aligned-unmapped.csv
-- DE-array
    -- _index
    -- deseq_tumorvsnormal.csv
    -- edger_tumorvsnormal.csv
    -- uqnorm_tumorvsnormal.csv
-- DE_heatmaps-document
    -- DE_heatmaps-heatmap_edger_tumorvsnormal-
heatmap.pdf
-- expr-matrix.csv
-- expr-normalizedArray
    -- _index
    -- deseqNormlzd.csv
    -- deseqPseudoNormlzd.csv
    -- edgerNormlzd.csv
    -- edgerPseudoNormlzd.csv
    -- uqnormNormlzd.csv
    -- uqnormPseudoNormlzd.csv
-- MDS-report.pdf
-- pipeline_configuration-currentStyles.csv
-- pipeline_configuration-topology.pdf
-- pipeline_statistics.csv
-- preprocess-QC1-reads
-- preprocess-QC1-report
    -- index.html
    -- Basic statistics
    -- Overrepresented sequences
    -- Quality control figures by figure type
    -- Quality control figures by sample
```

```
mirna_demo_report
-- All-html
    -- index.html
    -- pipeline_statistics.csv
    -- pipeline_component_instances.csv
    -- pipeline_topology.pdf
-- Module1_Preprocessing-html
    -- index.html
    -- preprocess_QC1_reads
    -- preprocess_QC1_report
        -- index.html
        -- Basic statistics
        -- Overrepresented sequences
        -- Quality control figures by figure type
        -- Quality control figures by sample
    -- aligned_pass.csv
    -- aligned_unmapped.csv
-- Module2_AlignmentQuantification-html
    -- index.html
    -- expr_matrix.csv
    -- expr_normalizedArray
        -- _index
        -- deseqNormlzd.csv
        -- deseqPseudoNormlzd.csv
        -- edgerNormlzd.csv
        -- edgerPseudoNormlzd.csv
        -- uqnormNormlzd.csv
        -- uqnormPseudoNormlzd.csv
    -- MDS_report.pdf
-- Module3_DifferentialExpression-html
    -- index.html
    -- DE_array
        -- _index
        -- deseq_tumorvsnormal.csv
        -- edger_tumorvsnormal.csv
        -- uqnorm_tumorvsnormal.csv
    -- DE_heatmaps_document
        -- DE_heatmaps-heatmap_edger_tumorvsnormal-
heatmap.pdf
```

Figure S4. Directory trees for the output of the miRNA pipeline (left) and the miRNA report (right).

```
rna_report
-- All-html
    -- index.html
-- Module1_Preprocessing-html
    -- index.html
    -- qc_mates
    -- qc_reads
    -- qc_report
-- Module2_AlignmentQuantification-html
    -- index.html
    -- BAM_alignment
    -- deseq_normalized_geneExpr_expr.csv
    -- Expression HTML report
    -- geneMatrix
    -- geneMatrix_FPKM_log2.csv
    -- geneMatrix_FPKM_table.csv
    -- STARoutput_folder
    -- transcriptMatrix_FPKM_log2.csv
    -- transcriptMatrix_FPKM_table.csv
-- Module3_DifferentialExpression-html
    -- index.html
    -- cuffdiff_folder
    -- diff_gene.csv
    -- res_pathwayGenes
    -- res_pathways.csv
-- Visuals-folder
    -- Heatmap of differentially expressed
genes_document.pdf
    -- MDS plots of expression_document.pdf
```

```
totalrna_report
-- All-html
    -- index.html
-- Module1_Preprocessing-html
    -- index.html
    -- qc_all_report
    -- qc_SRR1027172_report
    -- qc_mates_array
    -- qc_reads_array
-- Module2_AlignmentQuantification-html
    -- index.html
    -- BAM_alignment
    -- deseq_normalized_geneExpr_expr.csv
    -- exonMatrix
    -- Expression HTML report
    -- geneMatrix
    -- geneMatrix_FPKM_log2.csv
    -- geneMatrix_FPKM_table.csv
    -- STARoutput_folder
    -- transcriptMatrix_FPKM_log2.csv
    -- transcriptMatrix_FPKM_table.csv
-- Module3_DifferentialExpression-html
    -- index.html
    -- annovar_output
    -- cuffdiff_folder
    -- diff_exon.csv
    -- diff_gene.csv
    -- exome_variants
    -- res_pathwayGenes
    -- res_pathways.csv
-- Visuals-folder
    -- Heatmap of differentially expressed
genes_document.pdf
    -- MDS plots of expression_document.pdf
```

Figure S5. Directory trees of output reports for poly(A)-derived RNA and total RNA.

References

Bijian, K. *et al.* (2013). Targeting focal adhesion turnover in invasive breast cancer cells by the purine derivative reversine. *Br. J. Cancer*, **109**, 2810-2818.

Fernandez, P. L. *et al.* (1998). Cell cycle regulators and their abnormalities in breast cancer. *MP, Mol. Pathol.*, **51**, 305-309.

Gasco, M., Shami, S., Crook, T. (2002). The p53 pathway in breast cancer. *Breast Cancer Res.*, **4**, 70-76.

Genovesi, *et al.* (2011). Integrated analysis of miRNA and mRNA expression in childhood medulloblastoma compared with neural stem cells. *PLoS ONE* **6**, 23935.

Laxman, N. *et al.* (2015). Global miRNA expression and correlation with mRNA levels in primary human bone cells. *RNA* **21**, 1433-1443.

Luo, M., and Guan, J. L. (2010). Focal adhesion kinase: a prominent determinant in breast cancer initiation, progression and metastasis. *Cancer Lett.*, **289**, 127-139.

Williamson, V. *et al.* (2013). Detecting miRNAs in deep-sequencing data: a software performance comparison and evaluation. *Brief. Bioinformatics* **14**, 36-45.

Supplementary Tables

Table 1. KEGG pathways enriched with targets of differentially expressed miRNAs with FDR < 0.05.

Target genes were filtered for maximum correlation coefficient of -0.3 to their regulating miRNA, absolute Cuffdiff log2-fold change > 0.5, and Cuffdiff p-value < 0.05 between normal and tumor breast tissue. 'NDE' is the number of DEGs in a pathway with 'pSize' number of genes.

Pathway	NDE/pSize	p-value	FDR
Focal adhesion	41/192	9.80E-12	1.28E-09
Pathways in cancer	52/313	6.65E-11	4.36E-09
ECM-receptor interaction	21/81	1.06E-07	4.63E-06
Adipocytokine signaling pathway	16/67	3.46E-07	1.13E-05
Melanoma	16/69	3.02E-06	7.92E-05
Cell cycle	23/121	7.14E-06	0.000156
Progesterone-mediated oocyte maturation	18/82	1.81E-05	0.000338
Acute myeloid leukemia	13/52	5.98E-05	0.000974
Prostate cancer	17/84	7.39E-05	0.000974
PPAR signaling pathway	15/66	7.43E-05	0.000974
Tight junction	22/128	8.66E-05	0.001031
Oocyte meiosis	19/109	0.000254	0.002775
Small cell lung cancer	16/83	0.000316	0.003186
Pancreatic cancer	14/68	0.000459	0.004052
HTLV-I infection	32/255	0.000464	0.004052
Leukocyte transendothelial migration	18/114	0.000542	0.004434
Complement and coagulation cascades	13/66	0.000648	0.00496
Calcium signaling pathway	21/181	0.000682	0.00496
Cytokine-cytokine receptor interaction	28/260	0.001244	0.008575
Dopaminergic synapse	18/126	0.002059	0.012935
TGF-beta signaling pathway	7/82	0.002107	0.012935
Regulation of actin cytoskeleton	26/204	0.002172	0.012935
Cocaine addiction	10/49	0.002481	0.014131
Axon guidance	18/123	0.002619	0.014297
p53 signaling pathway	12/65	0.003011	0.015779
Transcriptional misregulation in cancer	22/166	0.003699	0.018638
Colorectal cancer	11/60	0.00409	0.019842
Renal cell carcinoma	12/66	0.004288	0.02006
Chronic myeloid leukemia	12/69	0.004843	0.021877
Aldosterone-regulated sodium reabsorption	7/38	0.007589	0.033138
Amoebiasis	15/105	0.008143	0.03441
Salivary secretion	11/87	0.01001	0.04098
Epithelial cell signaling in Helicobacter pylori infection	11/67	0.011945	0.047417

Table 2. Genes in Case I with splice variants having log2 fold changes in opposite directions in normal and tumor samples.

Transcript ID	Gene ID	Gene Name	Log2 fold-change	p-value
ENST00000331925	ENSG00000184009	ACTG1	3.06288	0.00085
ENST00000570382	ENSG00000184009	ACTG1	7.50747	0.042

ENST00000576214	ENSG00000184009	ACTG1	3.90393	0.04785
ENST00000615544	ENSG00000184009	ACTG1	-66.6478	0.0071
ENST00000421745	ENSG00000115760	BIRC6	-2.50422	0.0002
ENST00000471232	ENSG00000115760	BIRC6	3.03705	0.0104
ENST00000295926	ENSG00000163660	CCNL1	4.73393	0.0123
ENST00000483789	ENSG00000163660	CCNL1	-3.29165	0.0244
ENST00000309311	ENSG00000167658	EEF2	2.21651	0.00025
ENST00000598436	ENSG00000167658	EEF2	-4.28858	0.0345
ENST00000460638	ENSG00000100129	EIF3L	-16.7931	0.045
ENST00000477256	ENSG00000100129	EIF3L	2.50871	0.0041
ENST00000426059	ENSG00000115414	FN1	-9.84864	0.00415
ENST00000456923	ENSG00000115414	FN1	35.9734	0.00545
ENST00000472835	ENSG00000140718	FTO	4.29128	0.02365
ENST00000570395	ENSG00000140718	FTO	-3.38614	0.03585
ENST00000229239	ENSG00000111640	GAPDH	5.62051	0.0004
ENST00000396861	ENSG00000111640	GAPDH	-3.88432	0.0337
ENST00000504325	ENSG00000204628	GNB2L1	1.97066	0.02465
ENST00000506312	ENSG00000204628	GNB2L1	-100.481	0.02025
ENST00000509148	ENSG00000204628	GNB2L1	2.62999	0.02665
ENST00000511473	ENSG00000204628	GNB2L1	3.63035	0.0243
ENST00000329433	ENSG00000169045	HNRNPH1	5.05959	0.0326
ENST00000505087	ENSG00000169045	HNRNPH1	-3.78166	0.0351
ENST00000524552	ENSG00000109971	HSPA8	2.54595	0.0475
ENST00000528292	ENSG00000109971	HSPA8	-3.89067	0.03015
ENST00000530391	ENSG00000109971	HSPA8	3.68729	0.045
ENST00000534624	ENSG00000109971	HSPA8	4.4551	0.0448
ENST00000342160	ENSG00000086758	HUWE1	-2.00705	0.01795
ENST00000426907	ENSG00000086758	HUWE1	3.35019	0.01195
ENST00000381298	ENSG00000134352	IL6ST	1.34985	0.0415
ENST00000506241	ENSG00000134352	IL6ST	-3.23872	0.0315
ENST00000216181	ENSG00000100345	MYH9	1.24746	0.021
ENST00000401701	ENSG00000100345	MYH9	-74.7648	0.00915
ENST00000288235	ENSG00000157483	MYO1E	1.42455	0.04375
ENST00000559489	ENSG00000157483	MYO1E	-2.64487	0.0442
ENST00000412128	ENSG00000187514	PTMA	-6.68689	0.00845
ENST00000468027	ENSG00000187514	PTMA	5.07699	0.02095
ENST00000221975	ENSG00000105372	RPS19	-20.7288	0.04995
ENST00000598742	ENSG00000105372	RPS19	3.23795	0.023
ENST00000526248	ENSG00000149273	RPS3	-4.23444	0.0244
ENST00000527273	ENSG00000149273	RPS3	4.27252	0.01755
ENST00000301821	ENSG00000168028	RPSA	3.34381	0.0333
ENST00000444512	ENSG00000168028	RPSA	-3.68807	0.0188
ENST00000260356	ENSG00000137801	THBS1	1.54003	0.029
ENST00000397591	ENSG00000137801	THBS1	4.55162	0.00605
ENST00000559746	ENSG00000137801	THBS1	-2.60155	0.0398
ENST00000375224	ENSG00000127481	UBR4	3.86789	0.00355
ENST00000375254	ENSG00000127481	UBR4	-1.09662	0.0334

Table 3. Genes in Case II with splice variants having log2 fold changes in opposite directions in normal and tumor samples.

Transcript ID	Gene ID	Gene Name	Log2 foldchange	p-value
ENST00000464611	ENSG00000075624	ACTB	-1.4201	0.02355
ENST00000477812	ENSG00000075624	ACTB	2.54693	0.00395
ENST00000563987	ENSG00000149925	ALDOA	-3.60141	5.00E-005
ENST00000564546	ENSG00000149925	ALDOA	-2.28945	0.00025
ENST00000564595	ENSG00000149925	ALDOA	-2.55624	5.00E-005
ENST00000566012	ENSG00000149925	ALDOA	-2.35681	0.0005
ENST00000566130	ENSG00000149925	ALDOA	-2.03254	0.00155
ENST00000566846	ENSG00000149925	ALDOA	3.86603	0.0113
ENST00000569798	ENSG00000149925	ALDOA	-1.32858	0.0158
ENST00000354192	ENSG00000142192	APP	-3.02366	0.02965
ENST00000358918	ENSG00000142192	APP	2.3783	0.00055
ENST00000558401	ENSG00000166710	B2M	-1.69214	0.0008

ENST00000561139	ENSG00000166710	B2M	2.4074	0.0001
ENST00000545507	ENSG00000172270	BSG	-2.33338	0.00055
ENST00000612317	ENSG00000172270	BSG	1.64355	0.02555
ENST00000507081	ENSG00000145741	BTF3	4.09423	0.00365
ENST00000510787	ENSG00000145741	BTF3	1.83681	0.03695
ENST00000514505	ENSG00000145741	BTF3	-3.49397	0.02895
ENST00000535233	ENSG00000159403	C1R	1.16016	0.02765
ENST00000602480	ENSG00000159403	C1R	-7.15749	0.02075
ENST00000361675	ENSG00000122786	CALD1	2.24841	0.00245
ENST00000462181	ENSG00000122786	CALD1	-1.42901	0.0021
ENST00000477244	ENSG00000160014	CALM3	-2.90684	0.02325
ENST00000599839	ENSG00000160014	CALM3	2.52756	0.04715
ENST00000548898	ENSG00000135404	CD63	-5.02584	0.0015
ENST00000552754	ENSG00000135404	CD63	1.99498	0.0125
ENST00000308162	ENSG00000172757	CFL1	-1.08318	0.0037
ENST00000525451	ENSG00000172757	CFL1	-1.56703	0.0013
ENST00000531413	ENSG00000172757	CFL1	3.33807	0.04575
ENST00000519472	ENSG00000120885	CLU	1.10202	0.01815
ENST00000520796	ENSG00000120885	CLU	5.85211	0.01225
ENST00000522502	ENSG00000120885	CLU	-5.35161	0.01775
ENST00000412431	ENSG00000113712	CSNK1A1	-1.34512	0.0457
ENST00000606719	ENSG00000113712	CSNK1A1	2.29212	0.04485
ENST00000521368	ENSG00000044115	CTNNA1	2.21764	0.0137
ENST00000522792	ENSG00000044115	CTNNA1	-2.0519	0.04675
ENST00000408915	ENSG00000161249	DMKN	-4.19496	0.0005
ENST00000414866	ENSG00000161249	DMKN	1.36283	0.0224
ENST00000526531	ENSG00000177685	EFCAB4A	-2.20436	0.01225
ENST00000528315	ENSG00000177685	EFCAB4A	1.14888	0.0405
ENST00000221466	ENSG00000104870	FCGRT	1.17935	0.03235
ENST00000599701	ENSG00000104870	FCGRT	1.63017	0.004
ENST00000599988	ENSG00000104870	FCGRT	-3.71186	0.00415
ENST00000326324	ENSG00000077782	FGFR1	1.29853	0.0405
ENST00000356207	ENSG00000077782	FGFR1	1.61074	0.00565
ENST00000397103	ENSG00000077782	FGFR1	1.71124	0.00805
ENST00000397113	ENSG00000077782	FGFR1	-1.8207	0.0123
ENST00000344736	ENSG00000196924	FLNA	1.86532	0.01005
ENST00000498411	ENSG00000196924	FLNA	-643.334	0.0308
ENST00000503081	ENSG00000204628	GNB2L1	-3.07325	0.0099
ENST00000506312	ENSG00000204628	GNB2L1	2.37945	0.039
ENST00000507000	ENSG00000204628	GNB2L1	4.12157	0.00465
ENST00000511473	ENSG00000204628	GNB2L1	-1.14817	0.03925
ENST00000511566	ENSG00000204628	GNB2L1	-1.4889	0.0068
ENST00000509606	ENSG00000133835	HSD17B4	-1.8727	0.02115
ENST00000512644	ENSG00000133835	HSD17B4	1.45931	0.00985
ENST00000520216	ENSG00000133835	HSD17B4	1.91911	0.00395
ENST00000546856	ENSG00000135480	KRT7	1.40276	0.02155
ENST00000550153	ENSG00000135480	KRT7	-4.56792	0.02875
ENST00000478063	ENSG00000160789	LMNA	-2.37196	0.0281
ENST00000496738	ENSG00000160789	LMNA	1.57841	0.00755
ENST00000311604	ENSG00000130592	LSP1	-1.44345	0.04305
ENST00000405957	ENSG00000130592	LSP1	3.81978	5.00E-005
ENST00000485286	ENSG00000166508	MCM7	3.24024	0.00675
ENST00000489841	ENSG00000166508	MCM7	-1.50078	0.0152
ENST00000548580	ENSG00000092841	MYL6	-1.37054	0.02305

ENST00000549392	ENSG00000092841	MYL6	2.14585	0.0011
ENST00000296930	ENSG00000181163	NPM1	-1.68484	0.01055
ENST00000351986	ENSG00000181163	NPM1	-1.99037	0.00085
ENST00000518587	ENSG00000181163	NPM1	2.05859	0.0391
ENST00000523622	ENSG00000181163	NPM1	-1.4951	0.0025
ENST00000524204	ENSG00000181163	NPM1	-3.26325	0.02005
ENST00000570907	ENSG00000185624	P4HB	1.40108	0.02895
ENST00000574914	ENSG00000185624	P4HB	-2.07811	0.0002
ENST00000576380	ENSG00000185624	P4HB	1.98554	0.0249
ENST00000474281	ENSG00000102144	PGK1	2.10016	0.01475
ENST00000476531	ENSG00000102144	PGK1	-1.35396	0.0145
ENST00000389093	ENSG00000067225	PKM	-2.21651	0.00635
ENST00000561609	ENSG00000067225	PKM	3.06245	0.02695
ENST00000568883	ENSG00000067225	PKM	-3.78611	0.0116
ENST00000312419	ENSG00000175482	POLD4	-1.3826	0.01365
ENST00000539074	ENSG00000175482	POLD4	1.59476	0.02175
ENST00000392367	ENSG00000132341	RAN	1.17952	0.03975
ENST00000392369	ENSG00000132341	RAN	-1.69982	0.02165
ENST00000306256	ENSG00000157916	RER1	-3.10322	0.0029
ENST00000378512	ENSG00000157916	RER1	-1.52318	0.0119
ENST00000443438	ENSG00000157916	RER1	1.16916	0.0353
ENST00000559463	ENSG00000108107	RPL28	-1.52348	0.01175
ENST00000560055	ENSG00000108107	RPL28	1.28257	0.02495
ENST00000427905	ENSG00000100316	RPL3	-2.06024	0.03735
ENST00000453303	ENSG00000100316	RPL3	2.84266	0.04885
ENST00000394920	ENSG00000161016	RPL8	-2.40735	0.00395
ENST00000526668	ENSG00000161016	RPL8	-6.51315	0.00035
ENST00000528296	ENSG00000161016	RPL8	-1.64731	0.0147
ENST00000528957	ENSG00000161016	RPL8	3.07996	0.0054
ENST00000532702	ENSG00000161016	RPL8	1.45182	0.018
ENST00000449470	ENSG00000163682	RPL9	-1.26612	0.02685
ENST00000503040	ENSG00000163682	RPL9	3.17295	0.00535
ENST00000520627	ENSG00000008988	RPS20	-4.04906	0.0008
ENST00000618656	ENSG00000008988	RPS20	3.00943	0.0017
ENST00000510019	ENSG00000186468	RPS23	-1.08693	0.0219
ENST00000510210	ENSG00000186468	RPS23	1.65532	0.00855
ENST00000342503	ENSG00000139970	RTN1	-2.89289	0.0099
ENST00000611068	ENSG00000139970	RTN1	1.47291	0.02375
ENST00000371509	ENSG00000116171	SCP2	2.9063	0.001
ENST00000407246	ENSG00000116171	SCP2	1.54178	0.02605
ENST00000408941	ENSG00000116171	SCP2	-1.61131	0.0169
ENST00000430330	ENSG00000116171	SCP2	-1.17891	0.04625
ENST00000571041	ENSG00000167978	SRRM2	-1.62426	0.0178
ENST00000576674	ENSG00000167978	SRRM2	1.31037	0.0409
ENST00000400167	ENSG00000177426	TGIF1	-1.41699	0.0343
ENST00000549546	ENSG00000177426	TGIF1	2.10023	0.04345
ENST00000524659	ENSG00000154096	THY1	2.81945	0.0299
ENST00000524970	ENSG00000154096	THY1	-1.66632	0.00775
ENST00000267996	ENSG00000140416	TPM1	1.72781	0.0234
ENST00000334895	ENSG00000140416	TPM1	1.80981	0.0006
ENST00000558544	ENSG00000140416	TPM1	-2.98203	0.03105
ENST00000597136	ENSG00000130726	TRIM28	-1.43962	0.0349
ENST00000597172	ENSG00000130726	TRIM28	3.03835	0.03275
ENST00000598355	ENSG00000130726	TRIM28	-1.97788	0.01575

ENST00000397411	ENSG00000214063	TSPAN4	-1.1876	0.03705
ENST00000532375	ENSG00000214063	TSPAN4	1.08068	0.03925
ENST00000216185	ENSG00000100348	TXN2	-1.64723	0.0032
ENST00000411915	ENSG00000100348	TXN2	1.23356	0.01625

Table 4. Exon variants with a higher ratio in normal or tumor samples.
The 'diff_ratio' is positive when the alternative allele is higher in tumor than normal.

Chr	Start	End	Ref	Alt	Gene	ExonicFunc	dbSNP	Ratio
chr1	18481403	18481403	C	G	KLHDC7A	nonsynonymous_SNV	rs2992755	0.905
chr1	26031165	26031165	C	A	EXTL1	nonsynonymous_SNV	rs2736831	0.636
chr1	29148829	29148829	C	T	SRSF4	nonsynonymous_SNV	rs2230678	0.605
chr1	78628133	78628133	A	G	IFI44L	nonsynonymous_SNV	rs273259	0.747
chr1	93207166	93207166	A	C	CCDC18	nonsynonymous_SNV	rs2815413	0.71
chr1	102914362	102914362	G	A	COL11A1	nonsynonymous_SNV	rs3753841	0.642
chr1	109737079	109737079	C	T	GSTM3	nonsynonymous_SNV	-	0.623
chr1	157539235	157539235	C	T	FCRL5	nonsynonymous_SNV	rs2012199	0.948
chr1	200553438	200553438	G	C	KIF14	nonsynonymous_SNV	rs12120084	0.633
chr1	236249930	236249930	T	A	ERO1LB	nonsynonymous_SNV	rs2477599	0.766
chr10	58828793	58828793	T	C	BICC1	nonsynonymous_SNV	rs4948550	0.614
chr10	128119296	128119296	T	C	MKI67	nonsynonymous_SNV	rs2071498	0.622
chr11	10508192	10508192	G	A	MTRNR2L8	nonsynonymous_SNV	rs6484338	0.893
chr11	65021722	65021722	T	C	ARL2	nonsynonymous_SNV	rs664226	0.689
chr11	67634891	67634891	T	G	TBX10	nonsynonymous_SNV	rs3758938	0.705
chr11	67647021	67647021	C	T	ACY3	nonsynonymous_SNV	rs948445	0.74
chr11	71458501	71458501	G	C	NADSYN1	nonsynonymous_SNV	rs2276360	0.689
chr12	48004297	48004297	T	A	COL2A1	nonsynonymous_SNV	rs3803183	0.714
chr12	66305115	66305115	T	C	HELB	nonsynonymous_SNV	rs4430553	0.605
chr13	25301873	25301873	G	A	NUPL1	nonsynonymous_SNV	rs11556093	0.72
chr14	31395279	31395279	G	C	HEATR5A	nonsynonymous_SNV	rs7141532	0.618
chr15	40606445	40606445	G	C	CASC5	nonsynonymous_SNV	rs7177192	0.985
chr15	40621642	40621642	G	T	CASC5	nonsynonymous_SNV	rs2412541	0.87
chr15	40622992	40622992	A	G	CASC5	nonsynonymous_SNV	rs8040502	0.856
chr15	41699117	41699117	A	T	MGA	nonsynonymous_SNV	rs2178004	0.783
chr15	50182569	50182569	A	C	SLC27A2	nonsynonymous_SNV	rs1648348	0.936
chr15	63141567	63141567	G	A	LACTB	nonsynonymous_SNV	rs2729835	0.619
chr16	4883938	4883938	G	C	PPL	nonsynonymous_SNV	rs2037912	0.638
chr16	81022836	81022836	T	G	CENPN	stoploss	rs3743503	0.6
chr17	8048501	8048501	A	G	ALOX15B	nonsynonymous_SNV	-	0.6
chr17	39669745	39669745	C	T	PNMT	nonsynonymous_SNV	rs72554035	0.725
chr17	39727784	39727784	C	G	ERBB2	nonsynonymous_SNV	rs1058808	0.617
chr18	14779970	14779970	C	G	ANKRD30B	nonsynonymous_SNV	rs9675365	0.97
chr18	63503549	63503549	A	G	SERPINB5	nonsynonymous_SNV	rs1455555	0.685
chr19	4510548	4510548	C	T	PLIN4	nonsynonymous_SNV	rs7251858	0.921
chr19	43356099	43356099	G	A	CD177	nonsynonymous_SNV	rs199668750	1
chr19	43356103	43356103	T	G	CD177	nonsynonymous_SNV	rs200662237	1
chr19	43913925	43913925	C	T	ZNF45	nonsynonymous_SNV	rs407731	0.603
chr19	44329698	44329698	G	C	ZNF112	nonsynonymous_SNV	rs4280359	0.62

chr19	54278553	54278553	C	T	LILRB2	nonsynonymous_SNV	rs1128646	0.654
chr19	56088039	56088039	C	G	ZNF787	nonsynonymous_SNV	rs4077285	0.609
chr2	218644895	218644895	C	A	ZNF142	nonsynonymous_SNV	-	0.759
chr2	233841896	233841896	G	C	HJURP	nonsynonymous_SNV	rs3732215	0.754
chr20	38369011	38369011	C	T	LBP	nonsynonymous_SNV	rs2232613	0.6
chr20	45840651	45840651	G	A	SNX21	nonsynonymous_SNV	rs4638862	0.618
chr3	121489986	121489986	G	C	POLQ	nonsynonymous_SNV	rs3218649	0.642
chr3	165773492	165773492	C	T	BCHE	nonsynonymous_SNV	rs1803274	0.972
chr4	38828729	38828729	A	G	TLR6	nonsynonymous_SNV	rs5743810	0.812
chr4	40354405	40354405	A	G	CHRNA9	nonsynonymous_SNV	rs10009228	0.924
chr4	87811594	87811594	A	G	IBSP	nonsynonymous_SNV	rs13144371	0.635
chr4	177353596	177353596	C	T	NEIL3	nonsynonymous_SNV	rs13112358	0.684
chr4	177353681	177353681	A	C	NEIL3	nonsynonymous_SNV	rs13112390	0.824
chr5	80650052	80650052	G	A	MTRNR2L2	nonsynonymous_SNV	rs10942928	0.885
chr5	128349443	128349443	C	T	FBN2	nonsynonymous_SNV	rs154001	0.878
chr5	147906491	147906491	G	A	C5orf46	nonsynonymous_SNV	rs2250145	0.734
chr5	148095823	148095823	A	G	SPINK5	nonsynonymous_SNV	rs6892205	0.845
chr5	148097988	148097988	C	T	SPINK5	nonsynonymous_SNV	rs34482796	0.798
chr5	148100464	148100464	G	A	SPINK5	nonsynonymous_SNV	rs2303063	0.785
chr5	148101392	148101392	A	G	SPINK5	nonsynonymous_SNV	rs2303067	0.787
chr6	26107562	26107562	G	T	HIST1H1T	nonsynonymous_SNV	rs198845	0.969
chr6	26108054	26108054	C	G	HIST1H1T	nonsynonymous_SNV	rs198844	1
chr7	42048623	42048623	T	C	GLI3	nonsynonymous_SNV	rs846266	0.667
chr7	64928711	64928711	A	C	ZNF273	nonsynonymous_SNV	rs2017252	0.68
chr7	108194169	108194169	G	C	NRCAM	nonsynonymous_SNV	rs6958498	0.604
chr7	158167219	158167219	A	G	PTPRN2	nonsynonymous_SNV	rs1130495	1
chr8	117146964	117146964	C	T	SLC30A8	stopgain	-	0.648
chr9	137169863	137169863	C	A	LRRC26	nonsynonymous_SNV	rs7019671	0.64
chrX	15564175	15564175	T	C	ACE2	nonsynonymous_SNV	rs41303171	0.609
chrX	32362879	32362879	C	T	DMD	nonsynonymous_SNV	-	0.842
chrX	55090033	55090033	C	G	PAGE2	nonsynonymous_SNV	rs1845444	1
chrX	154013378	154013378	G	A	IRAK1	nonsynonymous_SNV	rs1059703	0.65

Table 5. KEGG pathways enriched with differentially expressed genes from Case I and with FDR < 0.05.
Genes were further filtered for absolute Cuffdiff log2-fold change > 0.5 'NDE' is the number of DEGs in a pathway with 'pSize' number of genes.

Pathway	NDE/pSize	p-value	FDR
Pathways in cancer	56/313	6.71E-15	8.93E-13
Osteoclast differentiation	34/125	3.12E-14	2.07E-12
HTLV-I infection	44/255	3.55E-11	1.58E-09
NF-kappa B signaling pathway	23/88	1.37E-09	4.56E-08
Cytokine-cytokine receptor interaction	31/260	2.44E-09	6.49E-08
Influenza A	31/160	5.72E-09	1.16E-07
p53 signaling pathway	18/65	6.12E-09	1.16E-07
MAPK signaling pathway	38/254	6.45E-08	1.04E-06
Cell cycle	25/121	7.04E-08	1.04E-06
Viral carcinogenesis	32/199	1.36E-07	1.81E-06
Acute myeloid leukemia	14/52	6.72E-07	8.13E-06
Transcriptional misregulation in cancer	28/166	8.53E-07	9.45E-06
Small cell lung cancer	18/83	1.45E-06	1.49E-05

Focal adhesion	29/192	1.89E-06	1.79E-05
Legionellosis	14/50	2.84E-06	2.52E-05
Measles	22/125	3.18E-06	2.64E-05
Chemokine signaling pathway	26/185	5.62E-06	4.40E-05
Amoebiasis	20/105	6.92E-06	5.11E-05
Leukocyte transendothelial migration	20/114	9.06E-06	6.34E-05
Chagas disease (American trypanosomiasis)	19/104	1.16E-05	7.70E-05
Pertussis	15/73	1.57E-05	9.93E-05
Prostate cancer	17/84	1.78E-05	0.000108
Malaria	13/50	1.93E-05	0.000111
Colorectal cancer	12/60	2.13E-05	0.000118
Epstein-Barr virus infection	27/188	2.86E-05	0.000152
Leishmaniasis	14/67	3.72E-05	0.00019
Prion diseases	9/33	3.90E-05	0.000192
B cell receptor signaling pathway	15/73	4.09E-05	0.000194
ErbB signaling pathway	13/84	4.79E-05	0.00022
Apoptosis	15/86	7.01E-05	0.000311
Fanconi anemia pathway	10/48	0.000117	0.000502
Complement and coagulation cascades	12/66	0.000166	0.000692
ECM-receptor interaction	15/81	0.000217	0.000875
Tuberculosis	23/167	0.000278	0.001087
Herpes simplex infection	22/169	0.000376	0.00143
Toxoplasmosis	19/127	0.000405	0.001496
Staphylococcus aureus infection	9/48	0.00048	0.001725
Rheumatoid arthritis	14/86	0.000509	0.001781
Cocaine addiction	9/49	0.000672	0.002292
T cell receptor signaling pathway	16/105	0.000839	0.002791
Hepatitis C	18/127	0.00089	0.002887
NOD-like receptor signaling pathway	11/55	0.00115	0.003642
African trypanosomiasis	7/34	0.001317	0.004018
Pancreatic cancer	12/68	0.001329	0.004018
Jak-STAT signaling pathway	19/150	0.001459	0.004313
Neurotrophin signaling pathway	16/117	0.002168	0.006268
Arrhythmogenic right ventricular cardiomyopathy (ARVC)	11/70	0.002563	0.007253
Bladder cancer	8/38	0.004122	0.01142
Intestinal immune network for IgA production	6/43	0.004413	0.011977
Non-small cell lung cancer	9/51	0.005751	0.015296
Aldosterone-regulated sodium reabsorption	7/38	0.007433	0.019384
Melanogenesis	12/98	0.008385	0.021446
Toll-like receptor signaling pathway	13/98	0.009296	0.023327
Circadian rhythm - mammal	4/20	0.011759	0.028962
Systemic lupus erythematosus	10/122	0.012336	0.029832
Chronic myeloid leukemia	10/69	0.014167	0.033647
GnRH signaling pathway	10/96	0.018721	0.043682
Epithelial cell signaling in Helicobacter pylori infection	7/67	0.019371	0.043715
Oocyte meiosis	13/109	0.019534	0.043715
Renal cell carcinoma	9/66	0.019721	0.043715

Table 6. KEGG pathways enriched with differentially expressed genes from Case II and with FDR < 0.05.
Genes were further filtered for absolute Cuffdiff log2-fold change > 0.5 'NDE' is the number of DEGs in a pathway with 'pSize' number of genes.

Pathway	NDE/pSize	p-value	FDR
Cytokine-cytokine receptor interaction	73/260	1.57E-08	2.13E-06
Cell cycle	39/121	1.21E-06	8.20E-05
Transcriptional misregulation in cancer	48/166	4.54E-06	0.00019
Neuroactive ligand-receptor interaction	63/267	5.60E-06	0.00019
ECM-receptor interaction	29/81	7.99E-06	0.000217

Alcoholism	47/169	1.50E-05	0.00034
Systemic lupus erythematosus	36/122	6.22E-05	0.001208
PPAR signaling pathway	21/66	7.57E-05	0.001286
Pathways in cancer	64/313	0.000142	0.002146
Focal adhesion	45/192	0.000271	0.003687
Amoebiasis	25/105	0.000398	0.004794
Axon guidance	33/123	0.000423	0.004794
Malaria	18/50	0.000672	0.007026
Fanconi anemia pathway	16/48	0.000974	0.00946
Oocyte meiosis	28/109	0.001106	0.010031
Viral carcinogenesis	47/199	0.001262	0.01058
Glutamatergic synapse	31/121	0.001323	0.01058
Progesterone-mediated oocyte maturation	23/82	0.001816	0.013718
Calcium signaling pathway	36/181	0.002507	0.017945
Rheumatoid arthritis	23/86	0.003983	0.027073
Adipocytokine signaling pathway	15/67	0.00418	0.027073
Salivary secretion	21/87	0.007348	0.045426
Bladder cancer	13/38	0.007765	0.045916

Table 7. Alignment statistics of the small RNA read mapping module.
This tab-delimited output file 'aligned-pass.csv' was generated with the set of 8 miRNA samples used for Performance statistics. If any of the 8 samples failed to achieve minimum alignment requirements, an 'aligned-fail.csv' file would also be output. A full list of output files from the demo before compilation in the HTML report can be previewed at: http://anduril.org/sepia/reports/mirna_demo/

Key	File	Total Sequenced Reads	Trimmed+ Quality Reads	Mapped Reads to Genome	Aligned Reads	Percent_ mapped	Percent_ mapped_ from_ Trimmed
TCGA_E2_A10B_01A	/SePIA/sepia_mirna/ .. TCGA_E2_A10B_01A-picard/sorted.bam	6454535	3856386	3758671	3469716	92.31	89.97
TCGA_E2_A10C_01A	/SePIA/sepia_mirna/ .. TCGA_E2_A10C_01A-picard/sorted.bam	6818793	3979564	3819253	3385600	88.65	85.07
TCGA_E2_A10E_01A	/SePIA/sepia_mirna/ .. TCGA_E2_A10E_01A-picard/sorted.bam	4205943	2434861	2375698	2142113	90.17	87.98
TCGA_E2_A10F_01A	/SePIA/sepia_mirna/ .. TCGA_E2_A10F_01A-picard/sorted.bam	6923515	4331828	4250660	3906214	91.90	90.17
TCGA_A7_A0CE_11A	/SePIA/sepia_mirna/ .. TCGA_A7_A0CE_11A-picard/sorted.bam	7785485	6016964	5899845	5518746	93.54	91.72
TCGA_A7_A0D9_11A	/SePIA/sepia_mirna/ .. TCGA_A7_A0D9_11A-picard/sorted.bam	4731512	3097433	2977189	2684230	90.16	86.66
TCGA_A7_A0DB_11A	/SePIA/sepia_mirna/ .. TCGA_A7_A0DB_11A-picard/sorted.bam	4391365	3011764	2857058	2574684	90.12	85.49
TCGA_BH_A0B3_11B	/SePIA/sepia_mirna/ .. TCGA_BH_A0B3_11B-picard/sorted.bam	4420577	3071909	2962931	2709715	91.45	88.21
